# G-Quadruplex loops regulate PARP-1 enzymatic activation

**DOI:** 10.1093/nar/gkaa1172

**Published:** 2020-12-11

**Authors:** Andrea D Edwards, John C Marecki, Alicia K Byrd, Jun Gao, Kevin D Raney

**Affiliations:** Department of Biochemistry and Molecular Biology, University of Arkansas for Medical Sciences, Little Rock, AR 72205, USA; Department of Biochemistry and Molecular Biology, University of Arkansas for Medical Sciences, Little Rock, AR 72205, USA; Department of Biochemistry and Molecular Biology, University of Arkansas for Medical Sciences, Little Rock, AR 72205, USA; Winthrop P. Rockefeller Cancer Institute, University of Arkansas for Medical Sciences, Little Rock, AR 72205, USA; Department of Biochemistry and Molecular Biology, University of Arkansas for Medical Sciences, Little Rock, AR 72205, USA; Department of Biochemistry and Molecular Biology, University of Arkansas for Medical Sciences, Little Rock, AR 72205, USA; Winthrop P. Rockefeller Cancer Institute, University of Arkansas for Medical Sciences, Little Rock, AR 72205, USA

## Abstract

G-Quadruplexes are non-B form DNA structures present at regulatory regions in the genome, such as promoters of proto-oncogenes and telomeres. The prominence in such sites suggests G-quadruplexes serve an important regulatory role in the cell. Indeed, oxidized G-quadruplexes found at regulatory sites are regarded as epigenetic elements and are associated with an interlinking of DNA repair and transcription. PARP-1 binds damaged DNA and non-B form DNA, where it covalently modifies repair enzymes or chromatin-associated proteins respectively with poly(ADP-ribose) (PAR). PAR serves as a signal in regulation of transcription, chromatin remodeling, and DNA repair. PARP-1 is known to bind G-quadruplexes with stimulation of enzymatic activity. We show that PARP-1 binds several G-quadruplex structures with nanomolar affinities, but only a subset promote PARP-1 activity. The G-quadruplex forming sequence found in the proto-oncogene c-KIT promoter stimulates enzymatic activity of PARP-1. The loop-forming characteristics of the c-KIT G-quadruplex sequence regulate PARP-1 catalytic activity, whereas eliminating these loop features reduces PARP-1 activity. Oxidized G-quadruplexes that have been suggested to form unique, looped structures stimulate PARP-1 activity. Our results support a functional interaction between PARP-1 and G-quadruplexes. PARP-1 enzymatic activation by G-quadruplexes is dependent on the loop features and the presence of oxidative damage.

## INTRODUCTION

G-Quadruplex DNA (G4DNA) consists of guanine-rich sequences of four tracts of three or more guanine bases separated by a variable number of nucleotides. The guanines interact by Hoogsteen hydrogen bonding to form planar quartets that stack into unique molecular structures (1–3). The nucleotides separating the tracts of guanines form the loop regions. More than 700 000 G4DNA forming sequences have been identified in the human genome (4) and >10 000 folded G4DNA were identified in cells using a G4DNA-specific antibody (5). Interestingly, G4DNA is enriched at regulatory genomic regions (2,6). G4DNA shows a 6-fold enrichment within 1 kilobase upstream of the transcription start sites (TSS), a 9-fold enrichment near nuclease hypersensitive sites (NHS), and a 230-fold enrichment for regions that are both near NHS and within promoters (7). Notably, G4DNA is overrepresented in promoters of proto-oncogenes (2,8) with 66% having at least 1 promoter G4DNA (7). Significant proto-oncogenes that contain G4DNA within their promoters include VEGF, c-MYC, c-KIT and KRAS (1,7,9). The prominence of G4DNA-forming sequences at key regions of the genome leads to questions regarding what functions G4DNA serves in the cell.

The folding or unfolding of G4DNA has proven to be important in controlling replication and transcription of several proto-oncogenes, as well as translation of the corresponding mRNAs (2,5,10–12). Others have reported that G4DNA serves as signaling elements for oxidative stress (13,14). Guanines have the lowest redox potential among nucleotides (15,16); G4DNA is highly susceptible to oxidative damage (17). Indeed, G4DNA loops and 5′ core guanines are primary targets for oxidation (18). Damage within G4DNA folds is inefficiently repaired by glycosylases and lesions alter G4DNA stability (16,19,20). Such occurrences at regulatory sites in the genome would appear to foster deleterious outcomes for the cell. However, the ‘spare-tire hypothesis’ states that various genes with 5 guanine-tracks in their promoter can maintain their G4DNA fold while extruding products such as 8-oxoguanine (8-oxo-G) and apurinic sites out of the fold into a loop (15,18). These looped G4DNA have been shown to be targets for repair enzymes and transcription factors such as APE-1, promoting an interlinking of transcription and DNA repair of key genes required to respond to stress conditions (13,15).

Such evidence supports a role for an evolutionary-selected pressure to concentrate G4DNA at particular regions of the genome. Defining regulatory proteins that respond to G4DNA in the presence or absence of damage will provide further supportive evidence of regulatory roles for G4DNA in the cell.

Poly (ADP-ribose) polymerase-1 (PARP-1) functions in both DNA repair and gene regulation. PARP-1 recognizes various DNA lesions such as single-strand and double-strand breaks (21,22). In response to DNA breaks, PARP-1 synthesizes polymers of ADP-ribose (PAR) on itself and various repair enzymes initiating DNA repair pathways and/or cell death (23). PARP-1 also binds non-B form DNA with enzymatic activation, especially structures with unpaired loop regions (24–27). However, the function of PARP-1 activity in response to non-B form DNA is less well characterized. Some studies suggest that PARP-1 activity in response to non-B form DNA could be implicated in chromatin remodeling and gene expression in the cell (26). Indeed non-B form DNA promotes PARP-1 trans-PARylation of histones (26), which facilitates chromatin dissociation and allows DNA accessibility for transcription (28).

The genomic landscape of PARP-1 potentially overlaps regulatory sites linked with G4DNA. ChIP-microarrays and nucleosomal ChIP-seq both revealed that PARP-1 is enriched at actively transcribed promoters (28–30). Further, PARP-1 nucleosome midpoints were much higher near DNase I hypersensitive sites and regions flanking chromatin organizer CCCTC-binding factor sites (28). Since regions that are both near NHS and within promoters show a 230-fold enrichment of G4DNA elements compared to the rest of the genome (7), there is a potential overlap between PARP-1 and G4DNA at these sites. In support of this correlation, PARP-1 has been shown to bind G4DNA found at promoters that regulate proto-oncogenes c-MYC, c-KIT and KRAS *in vitro* (31–33). G4DNA-stabilizing ligands triggered recruitment of PARP-1 to telomeres *in vivo* (34). ChIP-qPCR revealed that PARP-1 associated with the GA-element (a G4DNA structure) of murine KRAS promoter in cells (32). Reporter assays have also revealed that PARP-1 knockout mouse embryonic fibroblasts show increased c-MYC expression upon reintroduction of PARP-1 (33).

PARP-1 is represented at regulatory sites linked to G4DNA and is a dual functioning protein having roles in gene regulation and DNA repair. Therefore, PARP-1 is appealing to investigate the function of G4DNA. Although, PARP-1 is known to bind and respond to G4DNA, it is necessary to understand structural elements that regulate the interaction between PARP-1 and G4DNA. G4DNA forms various topologies in the genome (9,10,35–39). In this report, we characterized PARP-1 binding to G4DNAs found in promoters of various proto-oncogenes and telomeres. We also investigated the catalytic activities of PARP-1 in response to these G4DNAs. Furthermore, we studied PARP-1 activities in response to a group of G4DNAs that varied in loop length, topology, and the presence of oxidative damage. Our studies have provided substantial evidence of G4DNA structural variations that can modulate PARP-1 binding and activation. The observations herein suggest a relationship between G4DNA structure and PARP-1 activity that has functional implications.

## MATERIALS AND METHODS

### Oligonucleotides

G4DNA oligonucleotides were synthesized (Integrated DNA Technologies, Coralville, IA), gel-purified (40), and resuspended in 10 mM Tris–Cl pH 7.5, 1 mM EDTA, 140 mM KCl, and 8 mM MgCl_2_. All single-strand and double-strand DNA oligonucleotides were resuspended in 10 mM HEPES pH 7.5 and 1 mM EDTA. Oligonucleotide sequences are listed in Tables 1, 2, and Supplementary Table S1.

**Table 1.** c-KIT loop mutations. Guanine runs are highlighted in bold. Loop mutations are underlined

Name	Sequence
c-KIT L2 PEG	5′ **GGG** C **GGG**/iSpC3//iSp18/**GGG** A **GGGG** A GG 3′
c-KIT L2-L1	5′ **GGG**CGCGA**GGG**C**GGG** A **GGGG** A GG 3′
c-KIT L2-L3	5′ **GGG** C **GGG**A**GGG**CGCGA**GGGG** AGG 3′
c-KIT L2 5-ade	5′ **GGG** C **GGG**AAAAA**GGG** A **GGGG** AGG 3′
c-KIT L2 5-thy	5′ **GGG** C **GGG**TTTTT**GGG** A **GGGG** AGG 3′

**Table 2.** VEGF and oxidized substrates. Superscript numbers denote the run of guanine residues. Guanines involved in the most stable G4DNA fold are shown in bold. Replacement of guanine with 8-oxo-G mutation is underlined

Name	Sequence
VEGF	5′ C G**GGG**^1^ C **GGG**^2^ CCGG **GGG**^3^ C **GGG**G^4^ TCCCGGC GGGG^5^ C 3′
VEGF core 7 8-oxo-G	5′ C G**GGG**^1^ C (**8-oxo-G)GG**^2^ CCGG **GGG**^3^ C **GGG**G^4^ TCCCGGC GGGG^5^ C 3′
VEGF core 14 8-oxo-G	5′ C G**GGG**^1^ C **GGG**^2^ CCGG **(8-oxo-G)GG**^3^ C **GGG**G^4^ TCCCGGC GGGG^5^ C 3′
VEGF loop 12 8-oxo-G	5′ C G**GGG**^1^ C **GGG**^2^ CC(8-oxo-G)G **GGG**^3^ C **GGG**G^4^ TCCCGGC GGGG^5^ C 3′
T20 8-oxo-G	5′ TTT TTT TTT T(8-oxo-G)T TTT TTT TTT 3′

### Oligonucleotide gel electrophoresis

Gel electrophoresis experiments were performed with 20% native gels (19:1 acrylamide:bis-acrylamide). To ensure G4DNA maintained intact during the run, the gel and 1× TBE running buffer (89 mM Tris Borate pH 8.3 and 2 mM Na_2_ EDTA) were supplemented with 20 mM KCl. The 2 nM radiolabeled oligonucleotides were prepared in 10 mM Tris, 1 mM EDTA, and with/without 140 mM KCl and 8 mM MgCl_2_. Oligonucleotides in 10 mM Tris and 1 mM EDTA were heated and flash cooled. Oligonucleotides in 10 mM Tris, 1 mM EDTA, and with 140 mM KCl and 8 mM MgCl_2_ were heated at 95°C and slowly cooled to room temperature. Samples were resolved by electrophoresis for 8 hours, visualized using a Typhoon Trio phosphorimaging system, and purity quantified using ImageQuant software (GE Healthcare).

### Circular dichroism of G4DNA oligonucleotides

G4DNA oligonucleotides were heated to 95°C and slowly cooled to room temperature. Folded G4DNA in the presence or absence of PARP-1 were confirmed and topology identified by circular dichroism (CD) using a J715 and J-1100 spectropolarimeter (JASCO, Easton, MD). CD was measured using a quartz cell optical path length of 1 mm with a scanning speed of 100 nm/min, and a response time of 1 second over a wavelength range of 200–350 nm. Data were recorded five times, averaged, smoothed, and baseline-corrected. The CD spectra of G4DNA in the presence of PARP-1 was characterized after a 36 minute incubation.

### Protein purification

PARP-1 was purified as described (41). The full length human PARP-1 pET28a bacterial expression vector was a kind gift from Dr John Pascal (41). To reduce catalytic activity during expression, 10 mM benzamide was added to all cultures and resuspended pellets. PARP-1 protein was purified using nickel sepharose affinity chromatography, followed by heparin affinity chromatography, and size exclusion chromatography (41). The nickel column was washed consecutively with a low salt wash buffer (25 mM HEPES pH 8.0, 500 mM NaCl, 0.5 mM TCEP, 20 mM imidazole, protease inhibitors) and high salt wash buffer (25 mM HEPES pH 8.0, 1 M NaCl, 0.5 mM TCEP, 20 mM imidazole, protease inhibitors). The protein was eluted with 25 mM HEPES pH 8.0, 500 mM NaCl, 0.5 mM TCEP, 400 mM imidazole, and protease inhibitors. The protein fractions were pooled and diluted to 375 mM NaCl. The proteins were loaded onto a 5 ml HiTrap Heparin column equilibrated with 50 mM Tris pH 7.0, 1 mM EDTA, 0.1 mM TCEP and 375 mM NaCl. Bound PARP-1 was washed and eluted with a 40 column volume gradient of 375 mM NaCl to 1 M NaCl with 50 mM Tris pH 7.0, 1 mM EDTA and 0.1 mM TCEP at 3 ml/min. Collected fractions were concentrated at 3200 × g for 30 minutes with 10 000 molecular weight cut-off conical filters. During concentration, the protein was washed with gel filtration buffer (25 mM HEPES, 150 mM NaCl, 1 mM EDTA and 0.1 mM TCEP). PARP-1 was passed over a size exclusion chromatography and eluted with gel filtration buffer. Fractions were aliquoted, snap-frozen with liquid nitrogen, and stored at −80°C (Supplementary Figure S1).

The *PNC1* gene was amplified from *Saccharomyces cerevisiae* S288C using the oligonucleotides 5′-GGGGTCTCAAGGTATGAAGACTTTAATTGTTGTTGATA-3′ (BsaI recognition site underlined) and 5′-GCCTCGAGTTATTTATCCACGACATTGATGTTGTGG-3′ (XhoI recognition site underlined). The PCR products and pSUMO vector were digested with XhoI and BsaI. The vector and PCR inserts were ligated and the plasmids were sequenced. Rossetta2(DE3) *Escherichia coli* cells were transformed with the plasmid. PNC1 expression was induced with 0.1 mM IPTG overnight at 18°C and 250 RPM. Cells were pelleted at 3993 x g for 10 minutes at 4°C. The cell pellet was resuspended in buffer A (50 mM Tris pH 8.0, 500 mM NaCl, 10 mM imidazole, 10% glycerol) with addition of 2 mM PMSF, 0.75 mg/ml lysozyme and 1 EDTA-free protease inhibitor tablet/50 ml (aprotinin, bestatin, E-64, leupeptin, AEBSF, pepstatin A, metalloproteases) (Pierce, Waltham, MA, USA) with stirring for 1 hour. The lysate was placed on ice followed by microfluidization. Lysate was centrifuged at 109 000 x g for 1 hour at 4°C.The supernatant was loaded onto a nickel-sepharose resin and was washed with buffer A. The protein was eluted with a gradient of buffer A2 (50 mM Tris pH 8.0, 500 mM NaCl, 40 mM imidazole and 10% glycerol) and buffer B (50 mM Tris pH 8.0, 500 mM NaCl, 400 mM imidazole and 10% glycerol). Pooled fractions were placed in a 10 000 molecular weight cut-off snake-skin dialysis bag and dialyzed against final storage buffer overnight and 4 hours with fresh buffer (20 mM Tris pH 8.0, 150 mM NaCl, 1.5 mM β-mercaptoethanol (βME) and 1% glycerol). Purified PNC1 was aliquoted, snap-frozen with liquid nitrogen, and stored at −80°C.

### Fluorescence anisotropy

PARP-1 binding to various DNA sequences was performed in a 96-well plate with PARP-1 assay buffer (25 mM HEPES pH 8.0, 140 mM KCl, 8 mM MgCl_2_, 50 μg/ml BSA, 0.02 mM TCEP, 4% glycerol and 11.4 mM βME) with serially diluted PARP-1 protein and 5 nM fluorescently labeled DNA oligonucleotides. The samples were incubated for 30 minutes at room temperature in the dark. Fluorescence polarization values were measured by using a Victor3 V Multilabel Plate Counter (PerkinElmer, Boston, MA) with 485 and 535 nm filters. Fluorescence polarization measurements were recorded at 25°C. Anisotropy was calculated and plotted against protein concentration using KaleidaGraph (Reading, PA, USA) and fit to the quadratic equation to obtain equilibrium dissociation constants (*K*_D_). Data analysis was performed from three independent experiments. Error bars indicate standard deviation of three independent experiments.

### PARP-1 auto-modification reactions

PARP-1 auto-modification reactions were performed at room temperature in 5 mM nicotinamide adenine dinucleotide (NAD^+^), PARP-1 assay buffer (25 mM HEPES pH 8.0, 140 mM KCl, 8 mM MgCl_2_, 50 μg/ml BSA, 0.02 mM TCEP, 4% glycerol, and 11.4 mM βME) using 1 μM PARP-1 with 1 μM DNA for 15 minutes. The reactions were quenched with 0.1 M EDTA in SDS-loading buffer (15.6 mM Tris, 100 mM EDTA, 2.5% glycerol, 0.2 M βME, 0.26% SDS, 0.001% bromophenol blue). The reaction mixtures were resolved by 10% SDS-PAGE and stained with Coomassie Blue. Coomassie stained protein that migrated more slowly than PARP-1 was assumed to be PARylated. Gels were scanned using LAS4000 imager (GE Healthcare). Auto-modification was quantified as a ratio of slow migrating protein (above the 116 kDa molecular weight marker) to total Coomassie stained PARP-1 protein using ImageQuant Software (GE Healthcare Life Sciences, Pittsburgh, PA, USA). Data analysis was performed from three independent experiments. The *P* values were calculated using an unpaired t-test. Error bars indicate standard deviation of three independent experiments.

### Thermal melting of G4DNAs

CD was measured as a function of temperature with a J-1100 spectrophotometer (JASCO, Easton, MD, USA) using a quartz cell of 1 mm optical path length. Ellipticities for melting curves were recorded at 265 nm (the λ of the maximum molar ellipticity) over a temperature range of 4–95°C at 1°C/min. The data points were plotted versus temperature. Nonlinear regression: log(inhibitor) versus response—variable slope (GraphPad, San Diego, CA, USA) was used to calculate the melting temperature (*T*_m_), which is the temperature at which 50% of the structure is unfolded.

### G4DNA unfolding trap assay

The 2 nM radiolabeled G4DNA in PARP-1 reaction buffer (25 mM HEPES pH 8.0, 140 mM KCl, 8 mM MgCl_2_, 50 μg/ml BSA, 0.02 mM TCEP, 4% glycerol and 11.4 mM βME) was incubated with 750 nm PARP-1 mixed with or without 5 mM NAD^+^ and 10 nM DNA trap complimentary to the G4DNA (Q trap). Reactions were quenched at increasing times with 100 mm EDTA, 0.5% SDS, 150 nM trap complimentary to G4DNA trap (C trap), and 500 nM 18-bp dsDNA protein trap. Samples were separated by 20% native PAGE (19:1 acrylamide:bis-acrylamide), visualized using a Typhoon Trio phosphorimaging system, and quantified using ImageQuant software (GE Healthcare). Samples were resolved by electrophoresis with 1× TBE running buffer. Data analysis was performed from two independent experiments. The fraction of unfolded G4DNA was determined according to the following equation where P_t_ represents trapped G4DNA (duplex), S_t_ represents G4DNA substrate, t represents the time for unfolding for a specific sample, and 0 represents the blank sample:

$$\begin{equation*}\frac{{\frac{{{{\rm{P}}_{\rm{t}}}}}{{{{\rm{S}}_{\rm{t}}} + {{\rm{P}}_{\rm{t}}}}} - \frac{{{{\rm{P}}_0}}}{{{{\rm{S}}_0} + {{\rm{P}}_0}}}}}{{1 - \frac{{{{\rm{P}}_0}}}{{{{\rm{S}}_0} + {{\rm{P}}_0}}}}}\end{equation*}$$

Error bars indicate standard deviation of two independent experiments.

### Western blot analysis

PARP-1 auto-modification samples were separated by 10% SDS-PAGE and transferred to PVDF membranes for Western blot analysis. The membranes were blocked with 5% nonfat dry milk in TBST (20 mM Tris–Cl pH 7.5, 150 mM NaCl, 0.1% Tween 20). Membranes were probed with rabbit polyclonal anti-PAR (Catalog number: ALX-210–890A-0100, Lot number: 08141818, Enzo Life Sciences, Farmingdale, NY, USA) and mouse monoclonal anti-PARP-1 antibody (Catalog number: sc-8007, Lot number: E1314, Santa Cruz Biotechnology, Dallas, TX, USA) dilutions in 1% nonfat dry milk in TBST. Proteins were detected using horseradish peroxidase-conjugated secondary antibodies and Amersham ECL Plus (GE Healthcare Life Sciences, Pittsburgh, PA, USA). Blots were imaged using a Bio-Rad ChemiDoc MP Imaging System (Bio-Rad, Hercules, CA, USA). PAR relative to PARP-1 protein was quantified and plotted as a ratio using ImageQuant software (GE Healthcare).

### PNC1-OPT assay

To measure NAD^+^ consumption during PARP-1 auto-modification, reactions were performed in a 96-well plate with one reaction in the absence of NAD^+^ to account for background fluorescence. Auto-modification reactions were performed as described above. However, instead of quenching the reactions with SDS-loading buffer, auto-modification reactions were quenched with 5 mM benzamide after 15 minutes. Purified yPnc1 enzyme was added at 20 μg/ml to the quenched reactions. The reactions were incubated at 37°C for 1 hour. During the incubation period, OPT developer reagent (10 mM ortho-pthalaldehyde (OPT), 30% ethanol and 10 mM dithiothreitol) was pre-warmed for at least 15 minutes at 37°C in the dark. Following the incubation period, 50 μl of OPT developer reagent was added to each reaction under dim light. The reactions were incubated at room temperature for 1 hour at 45 RPM on an orbital shaker. The plates were transferred to a plate reader (Biotek Synergy 4, Winooski, VT, USA) with monochromators set to excitation at 420 nm and emission at 450 nm. Using the arbitrary fluorescence unit (AFU) readings for each reaction, the net fluorescence was calculated for each sample by subtracting the fluorescence of the control reaction (−NAD^+^) from the experimental reaction, *F*(corrected) = *F*(sample) – *F*(−NAD^+^). The resulting value is proportional to the amount of nicotinamide produced during the auto-modification reaction and therefore the amount of NAD^+^ consumed. Serially diluted nicotinamide was used as a standard curve. Data analysis was performed from three independent reactions. The *P* values were calculated using an unpaired t-test. Error bars indicate standard deviation of three independent experiments.

## RESULTS

### PARP-1 binds G4DNA with structure specificity

**Figure 1.**
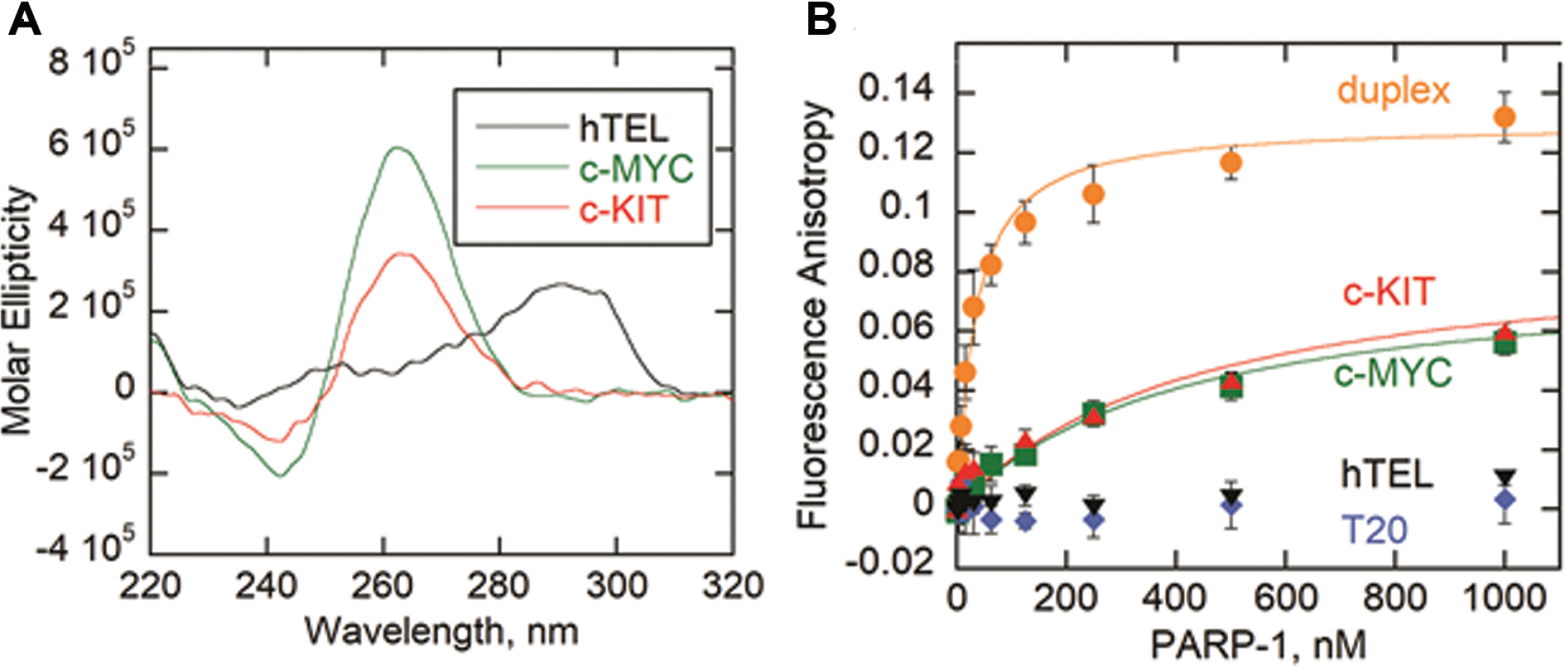
PARP-1 binds G4DNA with structure specificity. (**A**) CD spectra of 8.5 μM each of c-MYC, hTEL, and c-KIT oligonucleotides in the presence of 140 mM KCl and 8 mM MgCl_2_. Data were recorded five times, averaged, smoothed, and baseline-corrected. (**B**) Fluorescence anisotropy of 5 nM 18-bp dsDNA duplex, c-MYC G4DNA, c-KIT G4DNA, T20 ssDNA or hTEL G4DNA incubated with serially diluted PARP-1. Data were fit to the quadratic equation to obtain dissociation constants (*K*_D_). Error bars indicate standard deviation of 3 independent experiments.

We determined PARP-1 binding affinity to three G4DNA sequences: human telomeric DNA (hTEL), G4DNA from the promoter of the c-KIT proto-oncogene (42–44), and a G4DNA (Pu18 T14T23) modified from the promoter of the c-MYC proto-oncogene that folds into the preferred structure of the naturally occurring c-MYC G4DNA (45) (Supplementary Table S1). Signature CD spectra of G4DNA indicated folding with distinct patterns (46). CD spectra indicated proto-oncogenes c-KIT and c-MYC produce parallel G4DNA indicated by the peaks at ∼265 nm and the troughs at ∼240 nm (Figure 1A). The hTEL formed a hybrid G4DNA structure based on its CD spectrum (Figure 1A). To characterize PARP-1 affinity for G4DNA, equilibrium binding assays were performed with fluorescently labeled DNA oligonucleotides and purified PARP-1 (Supplementary Figure S1). Fluorescence anisotropy indicated that PARP-1 bound with nanomolar affinities to 18-bp dsDNA (*K*_D_ = 30 ± 10), c-KIT (*K*_D_ = 430 ± 60 nM) and c-MYC (*K*_D_ = 400 ± 50 nM), but showed little or no binding to hTEL or a single-stranded DNA, T20 (Figure 1B). PARP-1 appears to bind G4DNA with structural specificity, showing selectivity for parallel G4DNA compared to hybrid G4DNA.

### G4DNA loops regulate PARP-1 enzymatic activation

**Figure 2.**
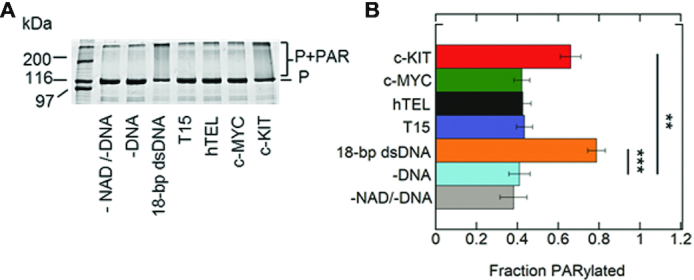
Parallel c-KIT stimulates PARP-1 activity. (**A**) ADP-ribosylation of PARP-1 (auto-modification) reactions were performed in the presence of 1 μM PARP-1 (P), 5 mM NAD^+^ and 1 μM DNA. Control experiments were performed in the absence of DNA and/or absence of NAD^+^. (**B**) Quantitation of auto-modification data in triplicate. Auto-modified PARP-1 was quantified as the smear above the 116 kDa molecular marker. The ratio of auto-modified PARP-1 to the sum of the total PARP-1 per lane was identified as fraction PARylated. The *P* values were calculated from three independent experiments using an unpaired t-test. (*) denotes two-sided *P*-value <0.05, (**) denotes two-sided *P*-value <0.01, (***) denotes two-sided *P*-value <0.001. Error bars indicate standard deviation of three independent experiments.

PARP-1 enzymatic activity leads to PARylation of proteins. PARP-1 is the major protein acceptor of ADP-ribose polymers (i.e. auto-modification) (21,47) that appears as a mass shift by SDS-PAGE. Gel-based auto-modification assays of PARP-1 were performed in the presence of DNA (Supplementary Table S1) and NAD^+^ (Figure 2A and B). While equilibrium binding assays indicated that PARP-1 bound to both parallel c-KIT and c-MYC (Figure 1B), only c-KIT stimulated PARP-1 activation. The quantity of PARylated-PARP-1 is not increased in the presence of NAD^+^ without DNA, or with ssDNA, hTEL or c-MYC.

It is possible that the c-KIT G4DNA structure undergoes structural distortions or changes in stability upon PARP-1 binding that promote enzymatic activity. CD spectroscopy was used to monitor these possible changes (Supplementary Figure S2). The CD spectra of c-KIT was monitored in 140 mM LiCl as a control. Li^+^ does not support G4DNA folding (4,48). In the presence of 140 mM KCl, PARP-1 does not unfold or remodel the c-KIT G4DNA structure when compared to c-KIT G4DNA alone. An experimental replicate provided similar results. PARP-1 accelerates the *in vitro* conversion of G4DNA to a transcriptionally more active B-form DNA in the presence of its complementary strand (33). DNA trap assays were also performed to monitor G4DNA stability in the presence of complimentary strand (Supplementary Figures S3–S5). The c-MYC G4DNA showed no unfolding in the presence of PARP-1 (Supplementary Figures S3A and S4). The c-KIT G4DNA showed some unfolding in the presence of complimentary strand over time (Supplementary Figures S3B, S5). However, this amount of unfolding was not increased in the presence of PARP-1. All G4DNA unfolding experiments were performed at protein concentrations above the *K*_D_ value. Therefore, G4DNA was saturated with PARP-1 protein under these experimental conditions.

**Figure 3.**
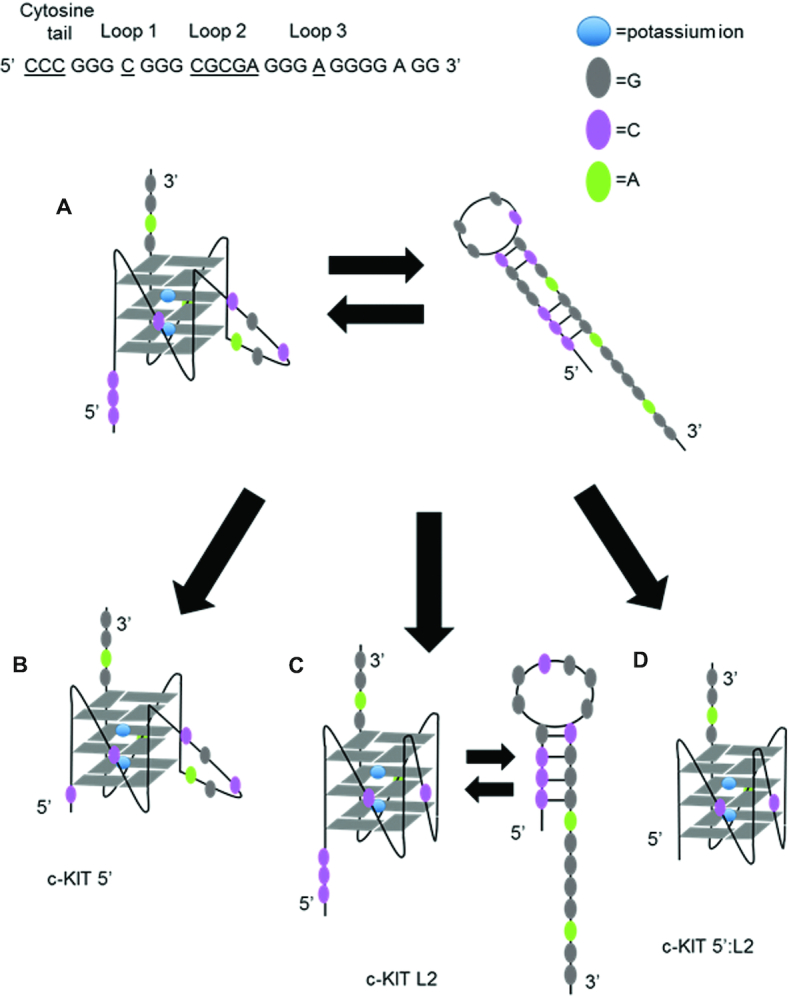
Proposed models of c-KIT DNA sequences. (**A**) c-KIT structural features include unpaired loop regions. The c-KIT G4DNA has five nucleotides in loop 2 and three cytosines at the 5′ end that can possibly equilibrate with stem-loop structures. (**B**) Shortening the cytosine tail at the 5′ end (c-KIT 5′) reduces stem-loop formation but maintains a pentanucleotide loop in the G4DNA structure. (**C**) Shortening the pentanucleotide loop (c-KIT L2) removes the unpaired loop region in G4DNA but stem–loop structures can still form. (**D**) Removing the three cytosines from the 5′-end and shortening the pentanucleotide loop simultaneously (c-KIT 5′:L2) removes all unpaired loop regions. The proposed structures of c-KIT and mutant constructs were identified using mfold Web Server (71).

PARP-1 enzymatic activity is stimulated by various non-B form DNA structures, such as cruciforms and stem loops, predominately at unpaired loop regions (26,27). We proposed that c-KIT G4DNA stimulates PARP-1 enzymatic activity due to its structure, which has unpaired loop regions (Figure 3A). Loop 2 of the folded c-KIT G4DNA is a pentanucleotide loop, separating tracts of guanines. In addition, the c-KIT sequence contains three cytosines at the 5′ end. These cytosines have the potential to base-pair with three guanines within the sequence, forming a smaller population of stem-loop structures such as the one presented in Figure 3A. Other gene regions similarly interchange between hairpins or G4DNA (49,50). However, G4DNA was the predominant structure in the presence of K^+^, as shown by NMR and CD spectroscopy (50). In our studies, CD spectroscopy similarly indicates that G4DNA is the predominant structure (Figure 1A).The appearance of c-KIT and c-MYC G4DNA was analyzed using a 20% native gel supplemented with 20 mM KCl to maintain G4DNA folding (Supplementary Figure S6). The major, fast running band of c-KIT DNA in 140 mM KCl and 8 mM MgCl_2_ represents 82% of the sample whereas the leading band for c-MYC DNA made up 97% of the sample. The c-KIT contains ∼18% other species that likely include stem-loops or intermolecular G4DNA, indicated by the slower moving, faint bands at higher molecular weight markers. Intramolecular G4DNA runs faster than its linear counterparts or intermolecular G4DNA aggregates (51,52).

**Figure 4.**
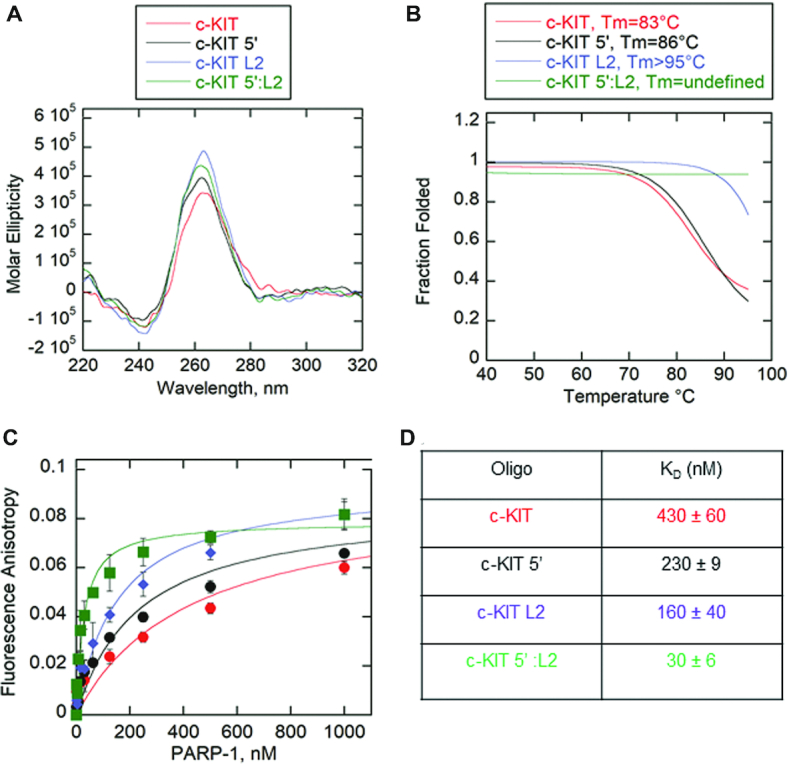
Removal of unpaired loops in c-KIT G4DNA results in tighter binding by PARP-1. (**A**) CD spectra of 8.5 μM of each modified c-KIT G4DNA in the presence of 140 mM KCl and 8 mM MgCl_2_. Data were recorded five times, averaged, smoothed, and baseline-corrected. CD spectra for c-KIT from Figure 1A was reproduced here for comparison. CD spectra of G4DNA indicates formation of parallel G4DNAs. (**B**) Thermal melting profile of modified c-KIT G4DNAs. Absorbance of circularly polarized light was measured at 265 nm across a temperature range of 4–95°C. Data was fit using a nonlinear regression: log (inhibitor) vs. response-variable slope (GraphPad) to calculate *T*_m_ at which 50% of G4DNA is melted. (**C**) Fluorescence anisotropy of 5 nM modified c-KIT incubated with serial dilutions of PARP-1. Fluorescence anisotropy for c-KIT from Figure 1B was reproduced here for comparison. Error bars indicate standard deviation of three independent experiments. (**D**) Data in panel C were fit to the quadratic equation to obtain *K*_D_ values.

**Figure 5.**
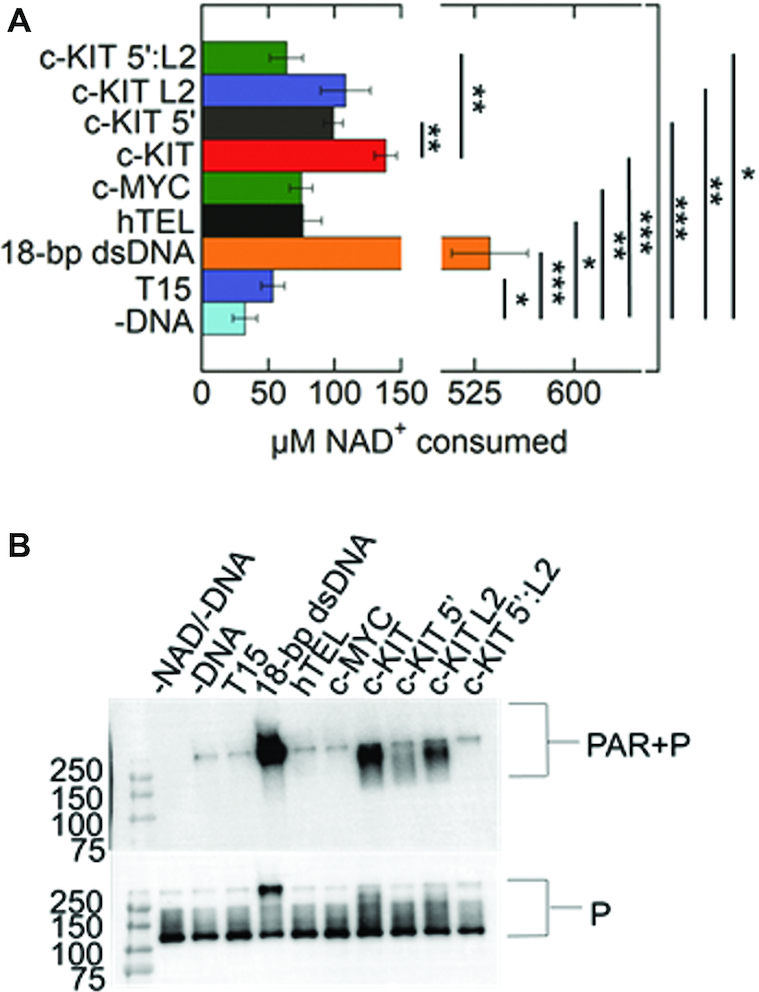
PARP-1 enzymatic activity is regulated by c-KIT loop features. (**A**) NAD^+^ consumption was measured using the PNC1-OPT assay. The auto-modification reactions were quenched with 50 mM benzamide after 15 minutes followed by addition of 20 μg/mL yPnc1. NAD^+^ consumed in the reaction is plotted for each form of DNA. The *P* values were calculated from three independent experiments using an unpaired *t*-test. (*) denotes two-sided *P*-value <0.05, (**) denotes two-sided *P*-value <0.01, (***) denotes two-sided *P*-value <0.001. Error bars indicate standard deviation of three independent experiments. (**B**) Western blot of PARP-1 auto-modification reactions. 1 μM PARP-1 (P) was incubated with 5 mM NAD^+^ and 1 μM DNA along with the assay mixture. Top panel is probed with an antibody specific for PAR. The bottom panel is probed for PARP-1, which serves as a loading control. Quantification of PAR relative to total PARP-1 provided in Supplementary Figure S9.

We modified the two features associated with the c-KIT sequence separately (i.e. c-KIT 5′, c-KIT L2) or combined (c-KIT 5′:L2) to determine whether these changes would reduce PARP-1 activation (Figure 3B–D) (Supplementary Table S1). The c-KIT 5′ loses its ability to form alternative DNA species such as stem-loops (Figure 3B). The central loop of c-KIT L2 is reduced from a pentanucleotide loop to a single-nucleotide loop, creating a stable G4DNA with short loops (Figure 3C). The CD spectra indicated that removal of the two features, separately or combined, resulted in parallel folds (Figure 4A). Thermal melting indicated that shortening the pentanucleotide loop, as in c-KIT L2 or c-KIT 5′:L2, increased the *T*_m_ to greater than 95°C (Figure 4B and Supplementary Figure S7). The melting curve for c-KIT 5′:L2 highlights significant stability supporting the observation that G4DNA stability is inversely correlated with loop length (53). We investigated changes in PARP-1 binding affinity for the c-KIT mutations (Figure 4C). Removing the pentanucleotide loop increased the affinity of PARP-1 for c-KIT. Interestingly, removing the 5′C tail had only a minor effect on the affinity, but removing both the pentanucleotide loop and the 5′C tail in c-KIT 5′:L2 resulted in an approximately 14-fold increase in affinity relative to c-KIT with no modifications (Figure 4D). This indicated an increased affinity between PARP-1 and G4DNA as the loop features were removed. However, this did not correlate with PARP-1 activity and in fact showed an inverse correlation. To quantify PARP-1 NAD^+^ consumption per oligo, we used the PNC1-OPT assay (54) (Supplementary Figure S8), which uses *Saccharomyces cerevisiae* nicotinamidase enzyme yPnc1 to convert PARylation by-product nicotinamide into a fluorescent molecule (54). PARP-1 is activated by 18-bp dsDNA and c-KIT, but less by T15, hTEL or c-MYC (Figure 5A). The activation mediated by c-KIT was 4-fold less than 18-bp dsDNA. Upon removal of the loop-forming features in either c-KIT L2 or c-KIT 5′, PARP-1 activation was reduced ∼1.3–1.4-fold. The c-KIT 5′:L2 reduced activation ∼2.2-fold, indicating that both the stem–loop and G4DNA loop features were necessary for activation of PARP-1 by c-KIT. A representative Western blot with PAR antibody similarly indicated that the 18-bp dsDNA and c-KIT promoted PARP-1 PARylation (Figure 5B and Supplementary Figure S9). Mutations of either the pentanucleotide loop or 5′C tail reduced activation. The combined mutations abolished PARP-1 activity.

**Figure 6.**
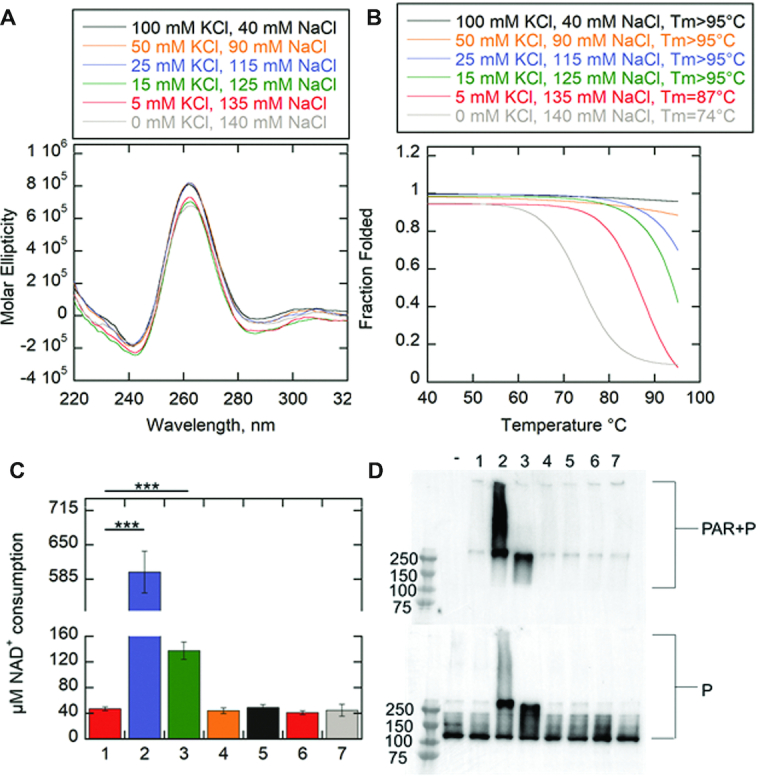
G4DNA stability has no impact on PARP-1 activity. (**A**) CD spectra of 8.5 μM c-KIT 5′:L2 in the presence of varying KCl and NaCl concentrations. Total monovalent cation concentration was 140 mM. The divalent cation from MgCl_2_ was held constant at 8 mM. Data were recorded five times, averaged, smoothed, and baseline-corrected. CD spectra of G4DNA indicates formation of parallel G4DNAs. (**B**) Thermal melting profile of c-KIT 5′:L2 as a function of varying monovalent salt concentrations. Absorbance of circularly light was measured at 265 nm across a temperature range of 4–95°C. Data was fit using a nonlinear regression: log (inhibitor) vs. response-variable slope (GraphPad) to calculate *T*_m_ at which 50% of G4DNA is melted. (**C**) NAD^+^ consumption was measured using the PNC1-OPT assay. The auto-modification reactions were quenched with 50 mM benzamide after 15 minutes followed by addition of 20 μg/ml yPnc1. NAD^+^ consumed in the reaction is plotted for each form of DNA. The *P* values were calculated from three independent experiments using an unpaired *t*-test. (*) denotes two-sided *P*-value <0.05, (**) denotes two-sided *P*-value <0.01, (***) denotes two-sided *P*-value <0.001. Error bars indicate standard deviation of three independent experiments. (**D**) Western blot of PARP-1 auto-modification reactions. 1 μM PARP-1 (P) was incubated with 5 mM NAD^+^ and 1 μM DNA along with the assay mixture. (−) minus NAD^+^/minus DNA in 140 mM KCl, 1.minus DNA in 140 mM KCl, 2. 10-bp dsDNA in 140 mM KCl, 3. c-KIT in 140 mM KCl, 4. c-KIT 5:L2 in 140 mM KCl, 5. c-KIT 5′:L2 in 100 mM KCl and 40 mM NaCl, 6. c-KIT 5′:L2 in 5 mM KCl and 135 mM NaCl, and 7. c-KIT 5′:L2 in 0 mM KCl and 140 mM NaCl. MgCl_2_ was held constant at 8 mM. Top panel is probed with an antibody specific for PAR. The bottom panel is probed for PARP-1, which serves as a loading control.

As previously mentioned, G4DNA stability is inversely correlated with loop length (53).To determine whether increased stability or lack of unpaired loops by c-KIT 5′:L2 led to a loss of PARP-1 activity, we decreased the stability of c-KIT 5′:L2 by changing monovalent cations in the buffer. All experiments thus far were performed in 140 mM KCl and 8 mM MgCl_2_. Cations stabilize G4DNA according to the following order: K^+^> Ca^2+^> Na^+^> Mg^2+^> Li^+^ and K^+^> Rb^+^> Cs^+^ (49,55). Therefore, c-KIT 5′:L2 was incubated in the presence of decreasing concentrations of potassium to lower its *T*_m_ to that of c-KIT. The total monovalent salt concentration of 140 mM was maintained by replacing potassium with the less stabilizing cation sodium. Magnesium concentration was held constant. In Figure 6A, c-KIT 5′:L2 maintained parallel G4DNA folds for all conditions with decreased potassium concentrations and increased sodium concentrations. However, the *T*_m_ decreased proportionally with reduced concentrations of potassium (Figure 6B and Supplementary Figure S10). *T*_m_ values could be defined for c-KIT 5′:L2 in 0 mM potassium and 5 mM potassium, at 74 and 87°C respectively. These *T*_m_ values were in the range of c-KIT which is 83°C (Figure 4B). The c-KIT 5′:L2 at 0, 5 and 100 mM KCl were used to investigate PARP-1 activity as a function of G4DNA stability. PARP-1 showed no activity for c-KIT 5:L2 at 0, 5 and 100 mM potassium concentrations, supporting the conclusion that loss of PARP-1 activity in response to c-KIT 5′:L2 was due exclusively to the removal of c-KIT loop features (Figure 6C and D). PARP-1 bound c-KIT 5′:L2 at 0 mM, 5 mM and 100 mM K^+^ with relatively similar affinities (Supplementary Figure S11).

**Figure 7.**
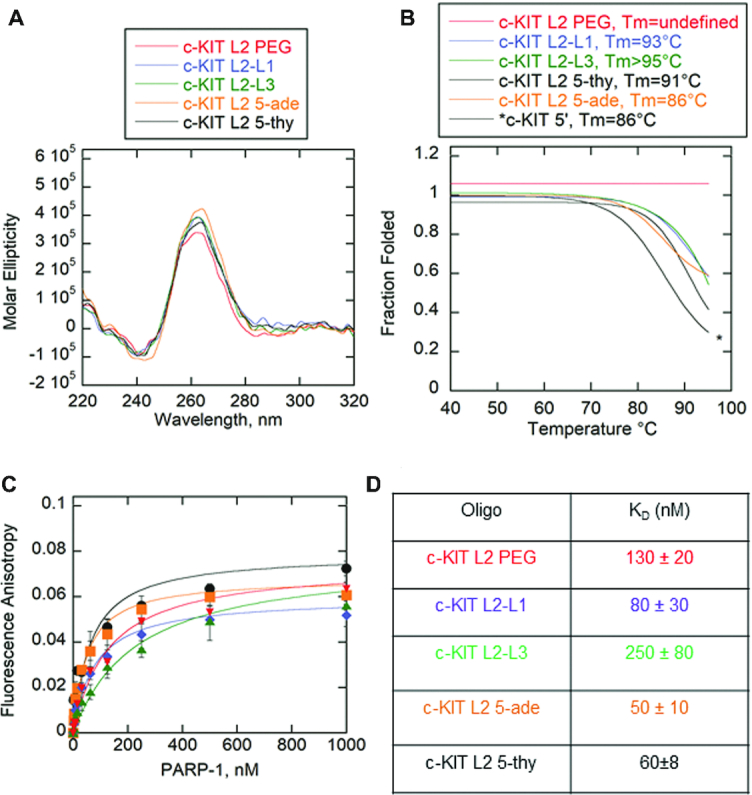
PARP-1 binds c-KIT G4DNA pentanucleotide loop mutants. (**A**) CD spectra of 8.5 μM of each c-KIT G4DNA with modified loops in the presence of 140 mM KCl and 8 mM MgCl_2_. Data were recorded five times, averaged, smoothed, and baseline-corrected. CD spectra indicate formation of parallel G4DNA. (**B**) Thermal melting profile of c-KIT G4DNA with modified loops. Absorbance of circularly polarized light was measured at 265 nm across a temperature range of 4−95°C. Data was fit using a nonlinear regression: log (inhibitor) versus response-variable slope (GraphPad) to calculate *T*_m_ at which 50% of G4DNA is melted. Thermal melting spectra for c-KIT 5′ from Figure 4B was reproduced here for comparison. (**C**) Fluorescence anisotropy of 5 nM c-KIT pentanucleotide loop mutants incubated with serial dilutions of PARP-1. Error bars indicate standard deviation of three independent experiments. (**D**) Data were fit to the quadratic equation to obtain *K*_D_ values.

The c-KIT substrate appears to regulate PARP-1 activity due to formation of two mixed species in solution: stem-loops and G4DNA with a long loop. In order to test the significance of PARP-1 activity exclusively to G4DNA loops, five c-KIT mutants were analyzed. The mutants lacked a 5′ cytosine tail and thus limit the potential to form stem-loops. The five c-KIT mutants differed in location of the pentanucleotide loop along the G4DNA fold, sequence of the pentanucleotide loop, and chemical composition of the loop (Table 1). To test whether the pentanucleotide loop in c-KIT G4DNA is a mediator of PARP-1 activity, the pentanucleotide loop was replaced with a combination of an 18-atom hexa-ethyleneglycol spacer (iSp18) and three successive ethyl groups (iSpC3) in c-KIT L2 PEG. The combined spacer modifications are equivalent to five nucleotides in length (Integrated DNA Technologies). Crystal structures of PARP-1 zinc fingers indicate PARP-1 binds with a bipartite mode to DNA structures (56). The DNA interaction engages a continuous region of the phosphodiester backbone and hydrophobic faces of nucleotide bases (56). If the pentanucleotide loop promotes PARP-1 activation, replacing the loop with polyethylene groups should abolish PARP-1 activity due to the absence of a phosphodiester backbone and nucleotide bases at this site. Also, the c-KIT pentanucleotide loop 2 was transferred to loops 1 and 3 to note whether location along the G4DNA structure impacted activity: c-KIT L2-L1 and c-KIT L2-L3, respectively. Loops 1 and 3 were switched to loop 2 in these respective mutations. To determine if the sequence of the loop impacts PARP-1 activity, the pentanucleotide loop 2 was replaced with either five adenines or five thymines: c-KIT L2 5-ade or c-KIT L2 5-thy respectively. All c-KIT mutants maintained a parallel G4DNA fold (Figure 7A). The *T*_m_ was measured for each substrate to determine whether the modifications affect overall G4DNA stability (Figure 7B and Supplementary Figure S12A). Previously, c-KIT 5′C was shown to have a *T*_m_ of 86°C (Figure 4B). The *T*_m_s for all c-KIT mutants were compared to c-KIT 5′C, since their 5′C tails were eliminated. With the exception of c-KIT L2 PEG, the mutations showed either the same or slightly higher melting profiles between 86°C and slightly >95°C (Figure 7B and Supplementary Figure S12A). The c-KIT L2 is more stable with the PEG spacer than DNA in the loop. Higher G4DNA stability in the presence of PEG spacers has been previously reported with telomeric G4DNA (57). However, we showed that stability does not correspond directly to PARP-1 binding or activation (Figure 6 and Supplementary Figure S11). In Figure 7C and D, PARP-1 bound all mutations with affinities ranging up to 5-fold tighter affinity than c-KIT 5′ (230 ± 9 nM from Figure 4C and D). There was no significant correlation in binding based on sequence, the presence of PEG, loop location, or G4DNA stability.

**Figure 8.**
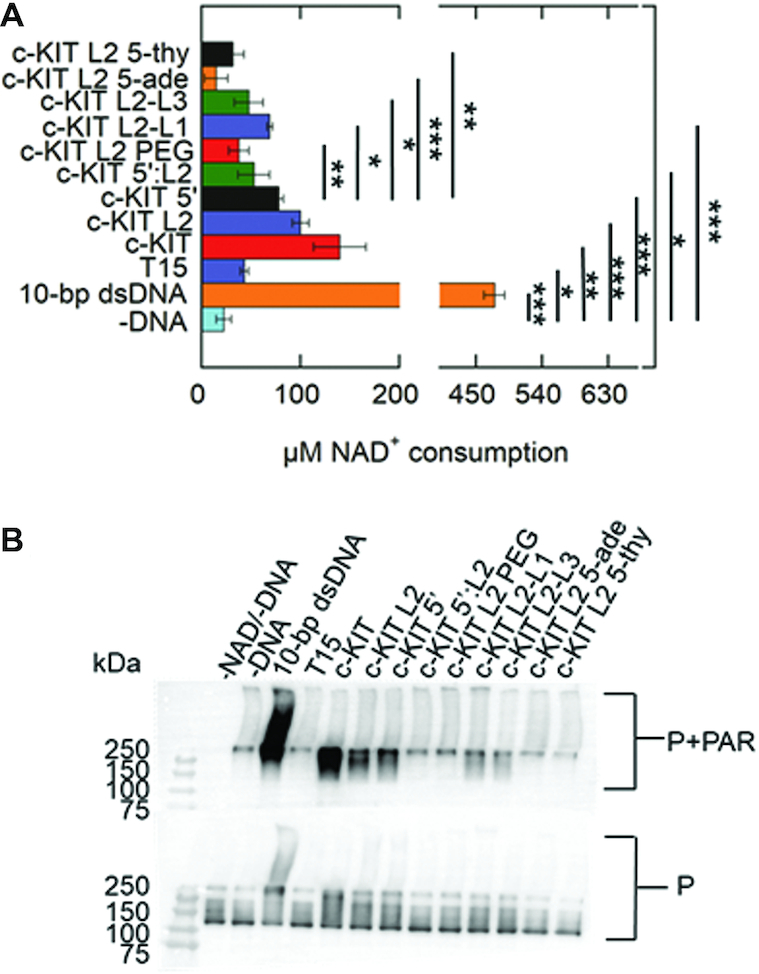
Impacts of G4DNA loop sequence, position, and chemical composition on PARP-1 activity. (**A**) NAD^+^ consumption was measured using the PNC1-OPT assay. The auto-modification reactions were quenched with 50 mM benzamide after 15 minutes followed by addition of 20 μg/ml yPnc1. NAD^+^ consumed in the reaction is plotted for each form of DNA. The *P* values were calculated from three independent experiments using an unpaired *t*-test. (*) denotes two-sided *P*-value<0.05, (**) denotes two-sided *P*-value <0.01, (***) denotes two-sided *P*-value <0.001. Error bars indicate standard deviation of three independent experiments. (**B**) Western blot of PARP-1 auto-modification reactions. 1 μM PARP-1 (P) was incubated with 5 mM NAD^+^ and 1 μM DNA along with the assay mixture. Top panel is probed with an antibody specific for PAR. The bottom panel is probed for PARP-1, which serves as a loading control. Quantification of PAR relative to total PARP-1 provided in Supplementary Figure S12B.

PARP-1 activity in response to c-KIT L2-L1 or c-KIT L2-L3 was ∼1.1- to 1.6-fold lower respectively compared to c-KIT 5′C (Figure 8A). PARP-1 activity in response to c-KIT L2 5-thy and c-KIT L2 5-ade was ∼2.4- to 5.2-fold lower than c-KIT 5′C and was relatively similar to the –DNA control, indicating that the moderate increase of PARP-1 activity in response to c-KIT pentanucleotide loop was completely due to its sequence. The c-KIT L2 PEG did not induce PARP-1 activity. The Western blot qualitatively suggests that c-KIT L2-L1 and c-KIT L2-L3 induce very moderate PARP-1 activity whereas c-KIT L2 PEG, c-KIT L2 5-ade, and c-KIT L2 5-thy did not induce PARP-1 activity (Figure 8B and Supplementary Figure S12B). The results are in agreement with the PNC1-OPT assay (Figure 8A).

### Oxidized G4DNA promotes significant PARP-1 enzymatic activity

**Figure 9.**
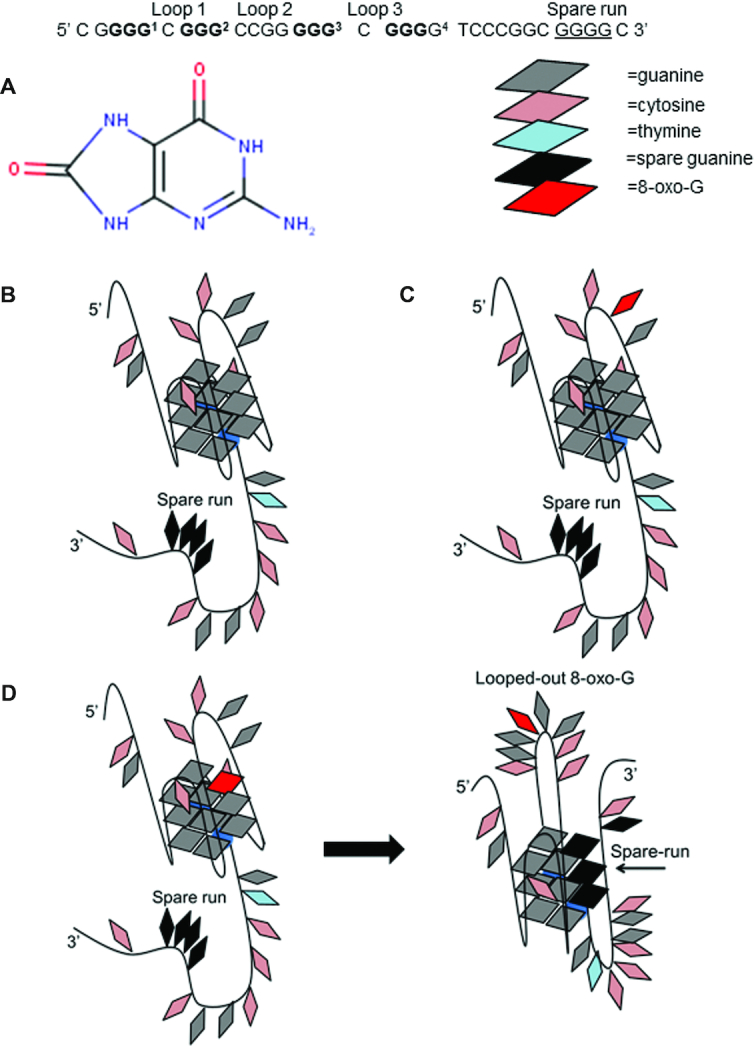
VEGF G4DNA models. Superscript numbers represent guanine runs. The most stable VEGF G4DNA consists of runs 1, 2, 3 and 4. Guanines involved in the most stable fold are in bold. The fifth spare guanine run is underlined. (**A**) Structure of 8-oxo-G lesion. (**B**) Proposed model of stable VEGF with 1,2,3,4-G4DNA forming group. (**C**) Proposed structure of VEGF loop 12 8-oxo-G. (**D**) Proposed structure of VEGF core 14 8-oxo-G and induced structural transition using the spare guanine run in black.

Our studies suggest that PARP-1 activity is regulated by some G4DNA loops. Therefore, PARP-1 may respond to oxidized G4DNA that transitions into looped structures to expose oxidized guanines for repair. The VEGF promoter has 5 runs of guanines where runs 1, 2, 3 and 4 have been shown to form the most stable G4DNA fold (Figure 9B) (Table 2) (58).

**Figure 10.**
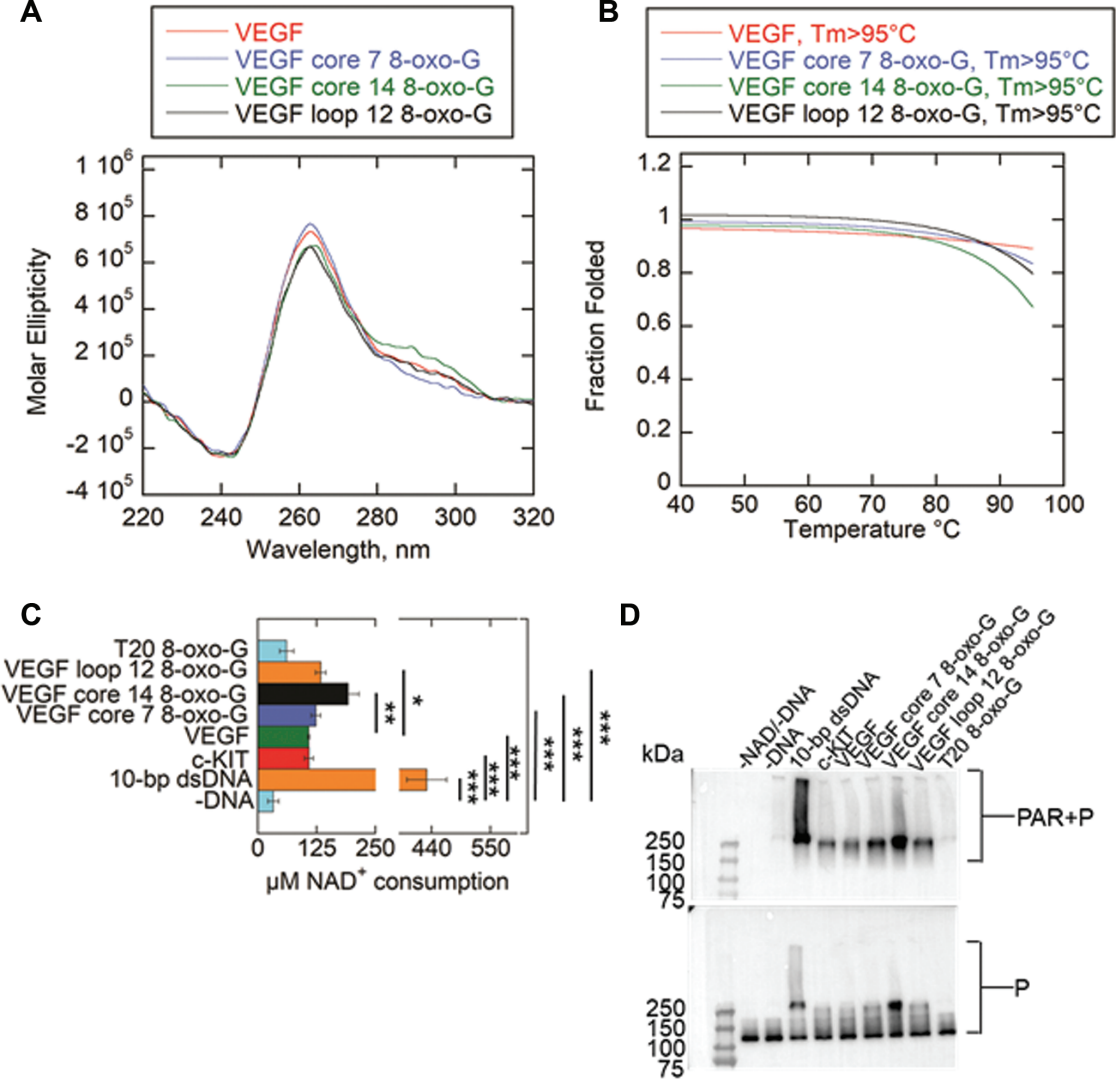
VEGF with 8-oxo-G at core guanines enhances PARP-1 activity. (**A**) CD spectra of 8.5 μM VEGF and 8-oxo-G substrates in the presence of 140 mM KCl and 8 mM MgCl_2_. Data were recorded five times, averaged, smoothed, and baseline-corrected. CD spectra of G4DNA indicates a mixture of parallel and hybrid G4DNA. (**B**) Thermal melting profile of VEGF substrates. Absorbance of circularly polarized light was measured at 265 nm across a temperature range of 4−95°C. Data was fit using a nonlinear regression: log (inhibitor) versus response-variable slope (GraphPad) to calculate *T*_m_ at which 50% of G4DNA is melted. (**C**) NAD^+^ consumption was measured using the PNC1-OPT assay. 1 μM PARP-1 (P) was incubated with 5 mM NAD^+^ and 1 μM DNA along with the assay mixture. The auto-modification reactions were quenched with 50 mM benzamide after 15 minutes followed by addition of 20 μg/ml yPnc1. NAD^+^ consumed in the reaction is plotted for each form of DNA. The *P* values were calculated from three independent experiments using an unpaired *t*-test. (*) denotes two-sided *P*-value <0.05, (**) denotes two-sided *P*-value <0.01, (***) denotes two-sided *P*-value <0.001. Error bars indicate standard deviation of three independent experiments. (**D**) Western blot of PARP-1 auto-modification reactions. 1 μM PARP-1 (P) was incubated with 5 mM NAD^+^ and 1 μM DNA along with the assay mixture. Top panel is probed with an antibody specific for PAR. The bottom panel is probed for PARP-1, which serves as a loading control. Quantification of PAR relative to total PARP-1 provided in Supplementary Figure S13B.

Core guanines 7 and 14, and loop guanine 12 were identified as some of the most susceptible oxidation sites in VEGF G4DNA, Figure 9 (18). Core guanines are in runs involved in stable G4DNA formation. Structural studies by the Burrows lab using DMS footprinting confirmed that VEGF uses the spare run 5 to maintain a G4DNA fold while looping out runs containing 8-oxo-G at core positions 7 or 14 (Figure 9D) (18). The presence of 8-oxo-G at loop guanine 12, which is not involved in core G4DNA runs, had no impact on the ability of VEGF to form its stable 1,2,3,4-fold (18) (Figure 9C). PARP-1 activity was monitored in response to VEGF G4DNA as well as VEGF G4DNA with 8-oxo-G lesions at loop guanine 12 and core guanines 7/14. Incorporating spare run 5 into G4DNA that are oxidized at core guanine runs should lead to the formation of alternatively, looped G4DNA structures that promote PARP-1 activity. The presence of 8-oxo-G at loop guanine 12 should have no impact on VEGF forming its native structure and therefore should not enhance PARP-1 activity. CD spectra indicated that all VEGF substrates with or without 8-oxo-G produce a peak ∼265 nm, a trough ∼240 nm, and a shoulder ∼295 nm (Figure 10A). This spectra indicates a mixture of parallel and hybrid G4DNA folds (18). Melting spectra were similar between VEGF substrates with *T*_m_ values >95°C (Figure 10B and Supplementary Figure S13A). Given a spare run, G4DNA can maintain similar stabilities in the presence of 8-oxo-G (18).

The PNC1-OPT assay and Western blotting were used to measure PARP-1 activity in response to VEGF and VEGF with 8-oxo-G (Figure 10C and D). Thymine-rich ssDNA with 8-oxo-G (T20 8-oxo-G) was used as a negative control. In Figure 10C, VEGF promoted similar levels of PARP-1 activity to c-KIT. This is possibly attributed to the fact that VEGF natively has a long loop of similar length to c-KIT. Guanines 12 and 13 do not participate in the most stable G4DNA fold (59).Therefore, loop 2 has two cytosines and two guanines, producing a tetranucleotide loop (Figure 9B). In addition, VEGF holds a cytosine run that may be involved in transient hairpins, similarly to c-KIT, Figure 9 (18). Using the PNC1-OPT assay, VEGF core 7 8-oxo-G and VEGF loop 12 8-oxo-G produced a small 1.1 to 1.2-fold increase in PARP-1 activity respectively. VEGF core 14 8-oxo-G produced a 1.8-fold increase in PARP-1 activity compared to VEGF (Figure 10C). T20 8-oxo-G did not induce PARP-1 activation. These results correlate with the representative Western blot (Figure 10D and Supplementary Figure S13B).

## DISCUSSION

The prominence of G4DNA in promoters of proto-oncogenes and other regulatory sites suggests that there are proteins that recognize these structures to regulate the chromatin and DNA structural landscape. We have evidence that suggests PARP-1 binds G4DNA in the absence of DNA lesions or breaks (Figure 1B). Our data demonstrated that PARP-1 bound parallel G4DNA structures. *In vitro*, PARP-1 did not bind hTEL which forms a hybrid G4DNA. Of 3087 G4DNA localized in the first 100 bases upstream of the TSS, 78% of them had at least one single-base loop (7). Analysis of G4DNA motifs in the human reference genome *in silico* also revealed an enrichment of small loop lengths (60). G4DNA with at least one single-base loop have a propensity to form parallel G4DNA (7). This suggests that proximal to the TSS, selective pressure has favored G4DNA with short, stabilizing loops and a predisposition towards a parallel structure (7,60). Given PARP-1 shows prominence at transcription regulatory regions (28,29) and our observations reveal that PARP-1 preferentially binds to parallel G4DNA we suggest that PARP-1 interacts with G4DNA at these sites in the genome. In addition, the concentration of PARP-1 in HeLa cells was found to be 2,030 nM (61). The concentration of PARP-1 is above our predicted *K*_D_ values for binding to G4DNA, indicating the possibility of interactions *in vivo*.

While our observations revealed PARP-1 binds to parallel G4DNAs, only a subset of G4DNAs promoted PARP-1 activation. The c-KIT G4DNA promoted PARP-1 activation (Figure 2). This activation was less than that mediated by an 18-bp dsDNA. This could be due to structural variations between c-KIT G4DNA and 18-bp dsDNA that differentially interact with PARP-1 DNA binding domains. Non-B DNA structures represent unequally favorable binding sites for PARP-1 to form catalytically active DNA-protein complexes (26). These differences would likely influence PARP-1 kinetic parameters (26). However, variable PARP-1 activity levels are functionally important (29,62). PARP-1 behaves as a ‘rheostat’ in the cell (23). Under the ‘PAR code’, PARP-1’s role in cellular processes depends on the duration, type, and strength of the stimuli, as well as the extent of PARylation (23,62). Hence, even low levels of PARP-1 activation appear to be biologically relevant.

PARP-1 accelerates the *in vitro* conversion of G4DNA to a transcriptionally more active B-form DNA in the presence of its complementary strand (33). In addition, PARP-1 promotes relaxation of supercoiled DNA (63). PARP-1 did not appear to affect G4DNA stability significantly upon binding or activation. CD spectroscopy of c-KIT G4DNA in the presence of PARP-1 did not show any significant changes in spectra in comparison to c-KIT alone (Supplementary Figure S2). In addition, DNA trap assays showed no significant increased unfolding of G4DNA in the presence of PARP-1 and complementary strands (Supplementary Figures S3-S5). Therefore, structural transitions were minor contributors to the observed PARP-1 activation.

Activation of PARP-1 by the c-KIT G4DNA is mediated by its DNA structural features (Figures 3 and 5). Loop 2 has a nucleotide length of 5, which may be an appropriate length to appear as unpaired loop region. Additionally, a small population of c-KIT may alternatively form stem-loops due to the cytosine-rich tail at the 5′ end (Figure 3 and Supplementary Figure S6). However, CD and NMR spectroscopy studies indicate G4DNA is the predominant structure in the presence of K^+^ in those gene regions that interchange between hairpins and G4DNA (50).Though these are separate structures, the evidence supports that G4DNA-mediated activation is dependent on loop regions. Eliminating these two loop-forming features (loop 2 or 5′C tail) separately lead to reduced PARP-1 activity (Figure 5). The removal of both loop-forming features together (c-KIT 5′:L2) led to diminished PARP-1 activation (Figure 5). Loss of PARP-1 activity could not be explained by increased G4DNA stability upon loop removal because lowering the stability of c-KIT 5′:L2 did not restore PARP-1 activity (Figure 6). These results favor the conclusion that the unpaired loop regions promoted PARP-1 activation. Further, replacing the c-KIT pentanucleotide loop with a PEG spacer (Table 1), which has similar length but lacking the deoxyribonucleosides, failed to activate PARP-1 (Figure 8). Our results provide further evidence that the pentanucleotide loop stimulated PARP-1 activity.

PARP-1 affinity for c-KIT G4DNA increased as the loop features were removed (Figure 4). Combining modified features on one c-KIT G4DNA construct (c-KIT 5′:L2) resulted in an ∼14-fold tighter binding affinity compared to non-mutated c-KIT G4DNA (Figure 4). Although PARP-1 bound c-KIT 5′:L2 with similar affinity to 18-bp dsDNA (*K*_D_ ∼ 30 nM), the c-KIT 5′:L2 G4DNA failed to activate PARP-1 (Figure 5). Thus, PARP-1 affinity for DNA substrates is not correlated with enzyme activity. Studies have reported that PARP-1 bound a host of RNA structures with variable affinities yet showed no enzymatic activation to any of the candidates (64). Once again, it is likely that G4DNA structural features dictate PARP-1 binding and activation.

Moving the pentanucleotide loop from loop 2 to loops 1 and 3 decreased PARP-1 activity (Figure 8). Changing the pentanucleotide sequence CGCGA to all adenines or thymines diminished PARP-1 activity compared to that of c-KIT 5′C (Figure 8). Interestingly, a PAR-CLIP followed by CLIP-seq identified that PARP-1 preferentially binds GC-rich residues, whereas AT-rich residues were depleted (65). In addition, other work identified that PARP-1 binds GC-rich nucleosomes (66). Given that the c-KIT pentanucleotide loop is GC-rich, our result indicates that PARP-1 activity in response to the loop was regulated by both structural and sequence elements.

G4DNA is variable in loop length, structure, and strand orientation (9). Therefore, it is possible that a host of other G4DNA, in addition to c-KIT G4DNA, promote PARP-1 activation in the absence of DNA damage. However, we suggest that these observations are biologically relevant. Oxidized G4DNA pose unusual, looped structures when damaged and are prime targets for an intertwining of DNA repair and transcription regulation (13,18). Our findings along with others identify base-excision repair (BER) proteins that respond to these looped structures. BER targets 8-oxo-G and other guanine lesions in double-strand DNA (16,67). However, less is known regarding the repair of oxidized G4DNA. PARP-1 and APE-1 are a part of the BER pathway and work together to sense DNA breaks and signal repair enzymes (67). Interestingly, recent reports indicate that APE-1 also stalls on looped, damaged G4DNA and promotes transcription activation (13,68). We show that PARP-1 responds to these looped G4DNA structures with enzymatic activation. Our results revealed that VEGF promotes PARP-1 activity similarly to c-KIT (Figure 10). This was expected due to VEGF G4DNA loop features (Figure 9). Placing 8-oxo-G at the 7th core guanine did not appear to enhance PARP-1 activity whereas 8-oxo-G at the 14th core guanine increased PARP-1 activity (Figure 10). The observation that VEGF core 7 8-oxo-G did not promote PARP-1 activation could be due to another level of structural complexity that remains to be determined. However, we show that placing 8-oxo-G in G4DNA can enhance PARP-1 activity. We believe that the observed PARP-1 activation in response to VEGF core 14 8-oxo-G was a result of structural changes to G4DNA caused by 8-oxo-G. The 8-oxo-G lesion has not been identified as being a strong PARP-1 activator. Apurinic/apyrimidinic sites and DNA breaks, which are generated following OGG-1 base-excision repair of 8-oxo-G, are considered the activating lesions for PARP-1 (69). OGG-1 increases the presence of oxidative stress-induced DNA strand breaks, leading to hyper-activation of PARP-1 (69). These signals were markedly lower in OGG-1 deficient cells (69). The observations herein suggest that there is an additional layer of complexity to the BER pathway. The BER pathway may target non-B form DNA structures that also require repair or epigenetic regulation to illicit stress responses. Future studies will investigate the involvement of PARP-1 with oxidized G4DNA in these cellular processes.

Looped G4DNA are biologically relevant. It has been reported that other cellular proteins preferentially bind long-looped G4DNA to promote molecular signaling (70). We conclude that the length, location, and sequence of G4DNA loops play a factor in PARP-1 activation upon binding to G-quadruplex structures. Undamaged G4DNA that display such loop features lead to moderate stimulation of PARP-1 activity. This modest increase in PARP-1 activity may be important in signaling, chromatin remodeling of histones, or transcription factor binding. Further, G4DNA with very short loops binds tightly to PARP-1 but does not stimulate PARP-1 activity. Oxidized G4DNA with a spare-run of guanines appears to increase PARP-1 activation compared to undamaged G4DNA. PARP-1 behaves as a rheostat in the cell (23). Depending on the stimuli, PARP-1 activity varies and regulates specific cellular responses (23). Large increases in PARP-1 activity in response to DNA damage may promote DNA repair responses and/or apoptosis. Our findings indicate an important biochemical interaction between dual-functioning PARP-1 and dual-functioning G4DNA, as potential gene expression regulators and responders to oxidative stress.

## DATA AVAILABILITY

The mfold Web Server (http://unafold.rna.albany.edu//?q=mfold/DNA-Folding-Form).

## Supplementary Material

gkaa1172_Supplemental_File
