# Supplementary material for: G-Quadruplex loops regulate PARP-1 enzymatic activation
Source: Nucleic Acids Res. 2020 Dec 11;49(1):416–31. doi: 10.1093/nar/gkaa1172 (PMC7797039; doi:10.1093/nar/gkaa1172)
Supplement: gkaa1172_Supplemental_File [file gkaa1172_supplemental_file.pdf]

G-Quadruplex Loops Regulate PARP-1 Enzymatic Activation

Andrea D. Edwards[1], John C. Marecki[1], Alicia K. Byrd[1,2], Jun Gao[1], and Kevin D. Raney[1,2*]
[1] Department of Biochemistry and Molecular Biology, University of Arkansas for Medical Sciences, Little Rock, Arkansas, 72205, USA
[2] Winthrop P. Rockefeller Cancer Institute, University of Arkansas for Medical Sciences, Little Rock, Arkansas, 72205, USA
To whom correspondence should be addressed. Tel: (501) 686-5244; Fax: (501) 686-8169; Email: RaneyKevinD@uams.edu.

Supplementary Data

Name	Sequence
T15	5’ TTT TTT TTT TTT TTT 3’
T20	5’ TTT TTT TTT TTT TTT TTT TT 3’
18-bp dsDNA	5’ GGG TTG CGG CCG CTT GGG 3’
	3’ CCC AAC GCC GGC GAA CCC 5’
10-bp dsDNA	5’ GCC GCT TGA G 3’
	3’ CGG CGA ACT C 5’
hTEL	5’ A GGG TTA GGG TTA GGG TTA GGG 3’
c-MYC*	5’ T GGG T GGG TA GGG T GGG T 3’
c-MYC* Q trap	5’ A CCC A CCC TA CCC A CCC A 3’
c-MYC* C trap	5’ T GGG T GGG TA GGG T GGG T 3’
c-KIT	5’ CCC GGG C GGG CGCGA GGG A GGGG A GG 3’
c-KIT Q trap	5’ CCT CCC CTC CCT CGC GCC CGC CCG GG 3’
c-KIT C trap	5’ CCC GGG C GGG CGCGA GGG A GGGG A GG 3’
c-KIT 5’	5’ __C GGG C GGG CGCGA GGG A GGGG A GG 3’
c-KIT L2	5’ CCC GGG C GGG __C__ GGG A GGGG A GG 3’
c-KIT 5’:L2	5’ ___ GGG C GGG __C__ GGG A GGGG A GG 3’

Supplementary Table 1. *denotes preferred structure of naturally occurring c-MYC G4DNA modified from the promoter of the c-MYC proto-oncogene.

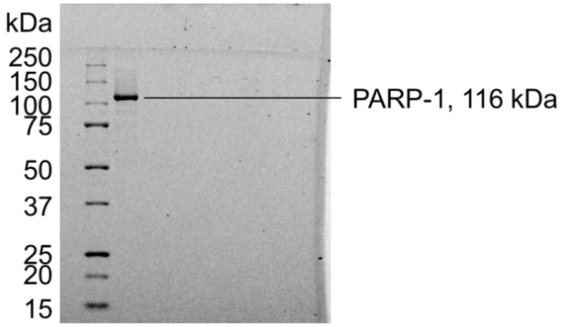

Figure S1. Purified recombinant PARP-1. His-tagged recombinant PARP-1 was expressed and purified using three chromatographic steps: Ni^{2+} affinity, heparin-sepharose, and gel filtration. Purified protein was stored in 25 mM Hepes pH 8, 150 mM NaCl, 1 mM EDTA, and 0.1 mM TCEP at -80ºC.

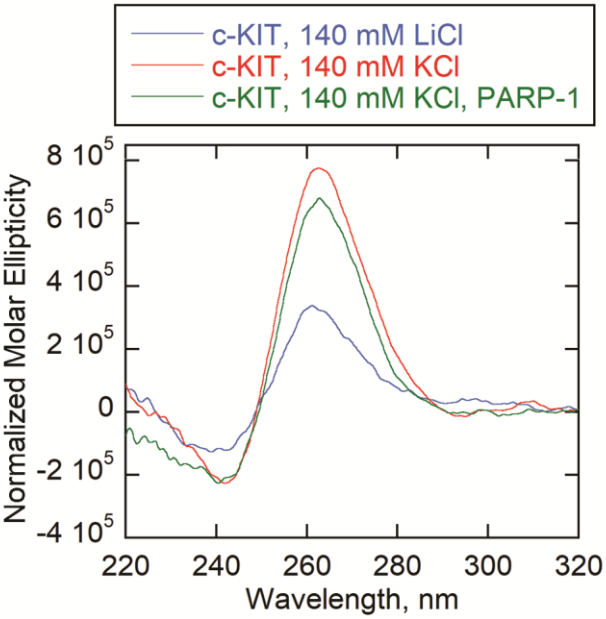

Figure S2. CD spectra of 5 µM c-KIT in the presence of 140 mM LiCl, 140 mM KCl, and 140 mM KCl and 5.5 µM PARP-1. PARP-1 was pre-incubated with c-KIT for 36 min. Data were recorded five times, averaged, smoothed, and baseline-corrected. CD spectra were normalized to c-KIT in 140 mM LiCl.

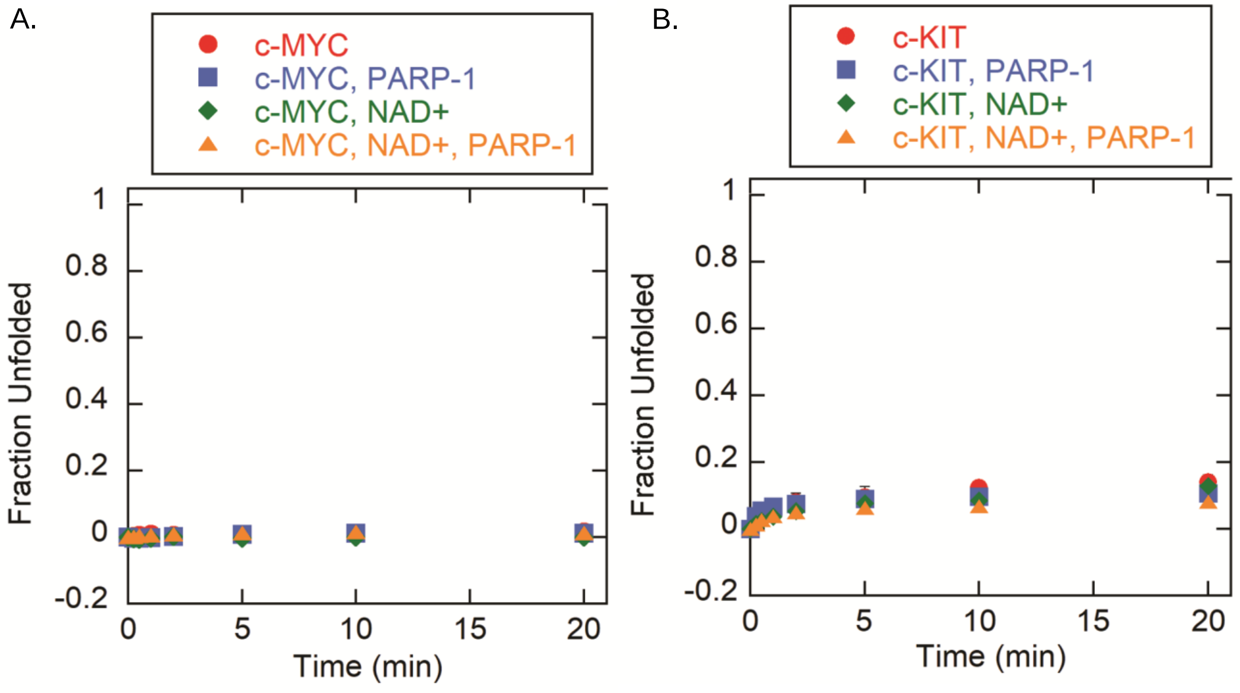

Figure S3. A. The 2 nM radiolabeled c-MYC was incubated alone or in the presence of 5 mM NAD^+, 750 nM PARP-1, or 5 mM NAD^+ and 750 nM PARP-1. All reactions were performed in the presence of 10 nM trap complimentary to c-MYC G4DNA (Q trap). B. The 2 nM radiolabeled c-KIT was incubated alone or in the presence of 5 mM NAD^+, 750 nM PARP-1, or 5 mM NAD^+ and 750 nM PARP-1. All reactions were performed in the presence of 10 nM trap complimentary to c-KIT G4DNA (Q trap). Reactions were quenched at increasing times with 100 mM EDTA, 0.5% SDS, 150 nM trap complimentary to G4DNA trap (C trap), and 500 nM 18-bp dsDNA protein trap. Fraction G4DNA unfolded was plotted versus time. Error bars represent the standard deviation of two separate experiments.

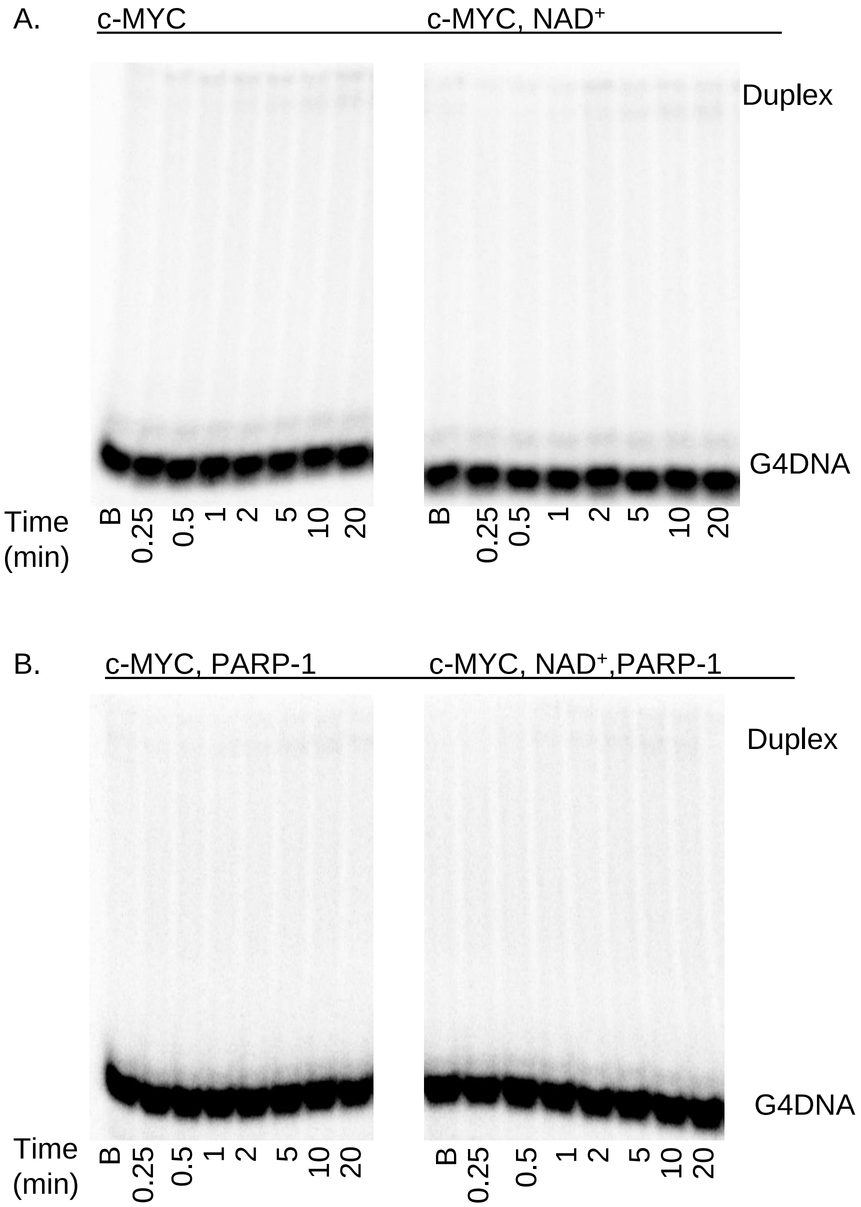

Figure S4. A. The 2 nM radiolabeled c-MYC was incubated with 10 nM trap complimentary to c-MYC G4DNA (Q trap) and with/without 5 mM NAD^+ for 0-20 min. B. The 2 nM radiolabeled c-MYC was incubated with 750 nM PARP-1, 10 nM trap complimentary to c-MYC G4DNA (Q trap), and with/without 5 mM NAD^+ for 0-20 min. Reactions were quenched at increasing times with 100 mM EDTA, 0.5% SDS, 150 nM trap complimentary to G4DNA trap (C trap), and 500 nM 18-bp dsDNA protein trap. Unfolded G4DNA trapped with Q trap represented as duplex. Reactions are shown on a 20% native gel (19:1 acrylamide:bis-acrylamide).

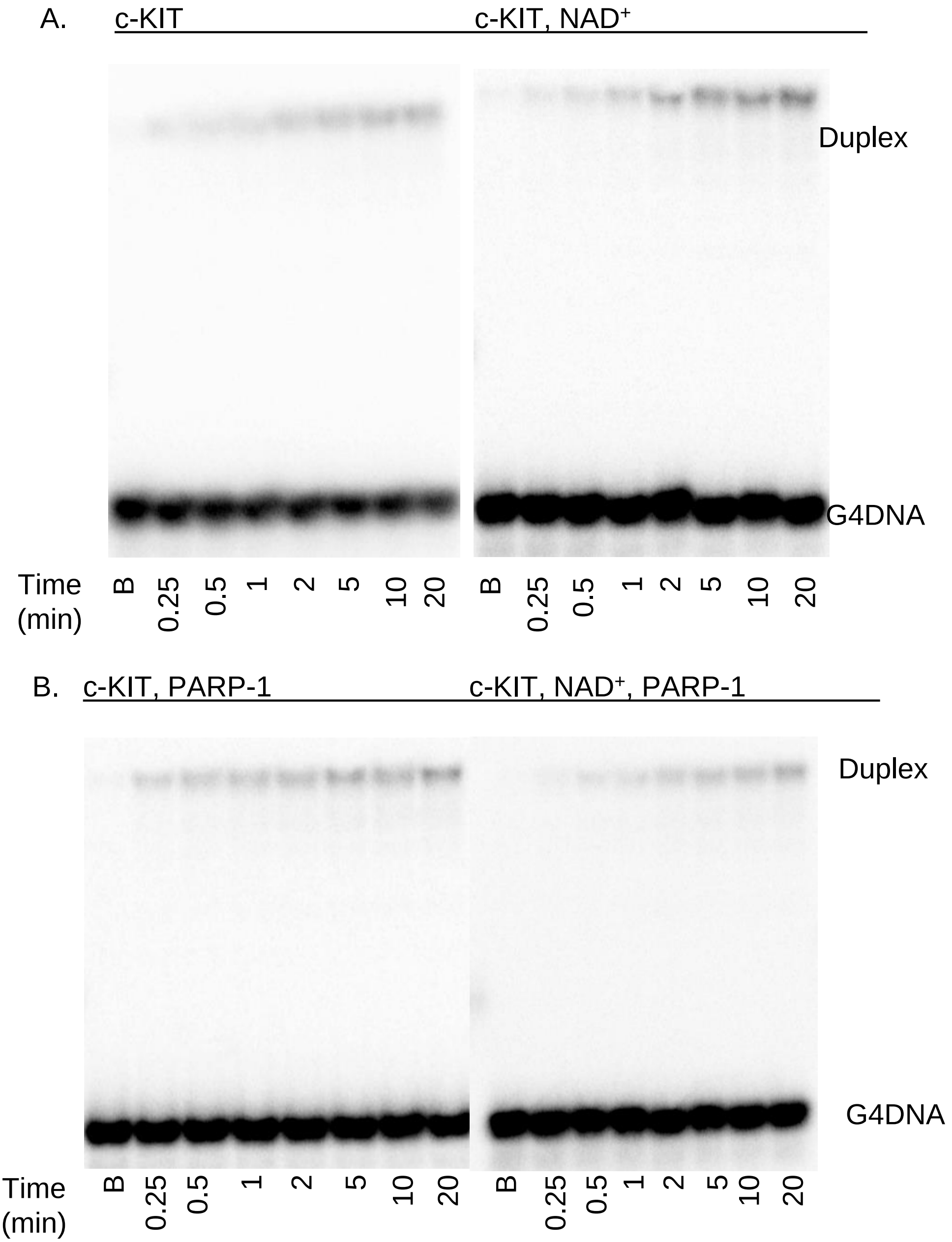

Figure S5. A. The 2 nM radiolabeled c-KIT was incubated with 10 nM trap complimentary to c-KIT G4DNA (Q trap) and with/without 5 mM NAD^+ for 0-20 min. B. The 2 nM radiolabeled c-KIT was incubated with 750 nM PARP-1, 10 nM trap complimentary to c-KIT G4DNA (Q trap), and with/without 5 mM NAD^+ for 0-20 min. Reactions were quenched at increasing times with 100 mM EDTA, 0.5% SDS, 150 nM trap complimentary to G4DNA trap (C trap), and 500 nM 18-bp dsDNA protein trap. Unfolded G4DNA trapped with Q trap represented as duplex. Reactions are shown on a 20% native gel (19:1 acrylamide:bis-acrylamide).

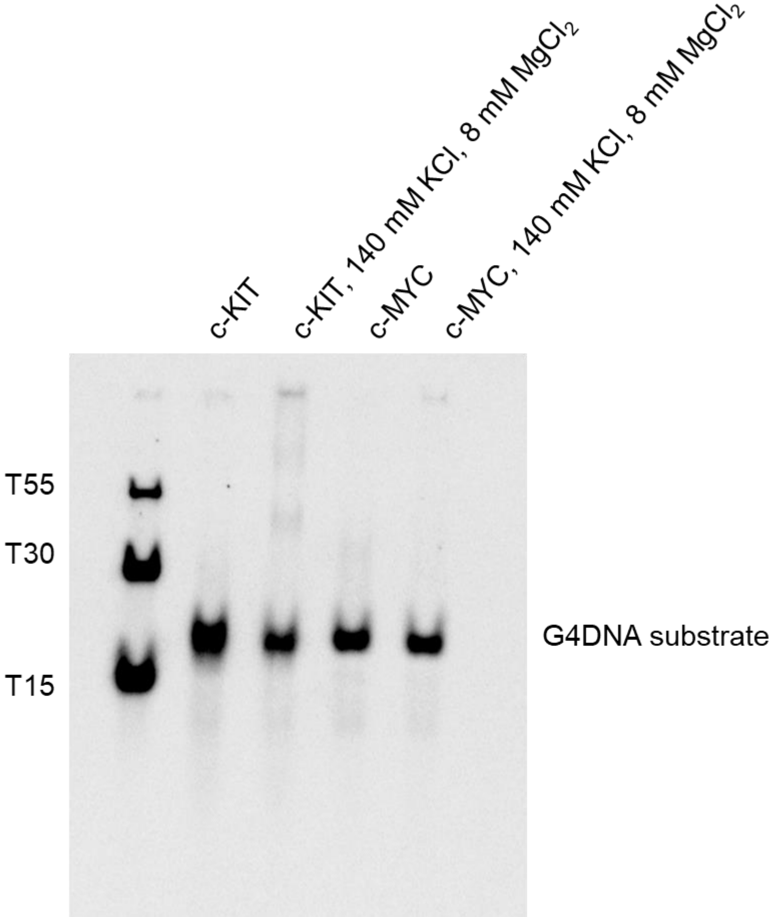

Figure S6. Oligonucleotide gel electrophoresis. Radiolabeled oligos were resuspended in 10 mM Tris-1 mM EDTA with/without 140 mM KCl and 8 mM $MgCl_2$. G4DNA in buffer were heated at 95°C and flash cooled. G4DNA in buffer and salt were heated at 95°C and slowly cooled to room temperature. Samples were resolved by electrophoresis on a 20% native gel supplemented with 20 mM KCl. The purities of the major DNA bands in 140 mM KCl and 8 mM $MgCl_2$ are as follows: c-KIT 82% and c-MYC 97%.

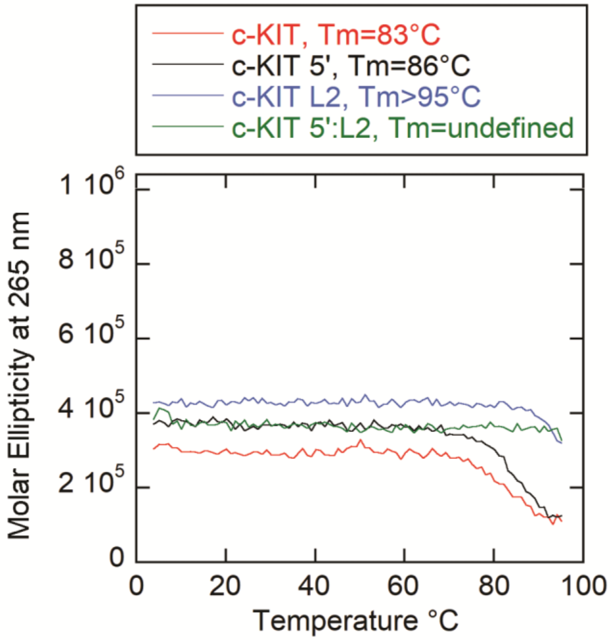

Figure S7. Raw molar ellipticity profile of c-KIT G4DNA mutants in Fig. 4B. Molar ellipticity was measured at 265 nm across a temperature range of 4-95ºC.

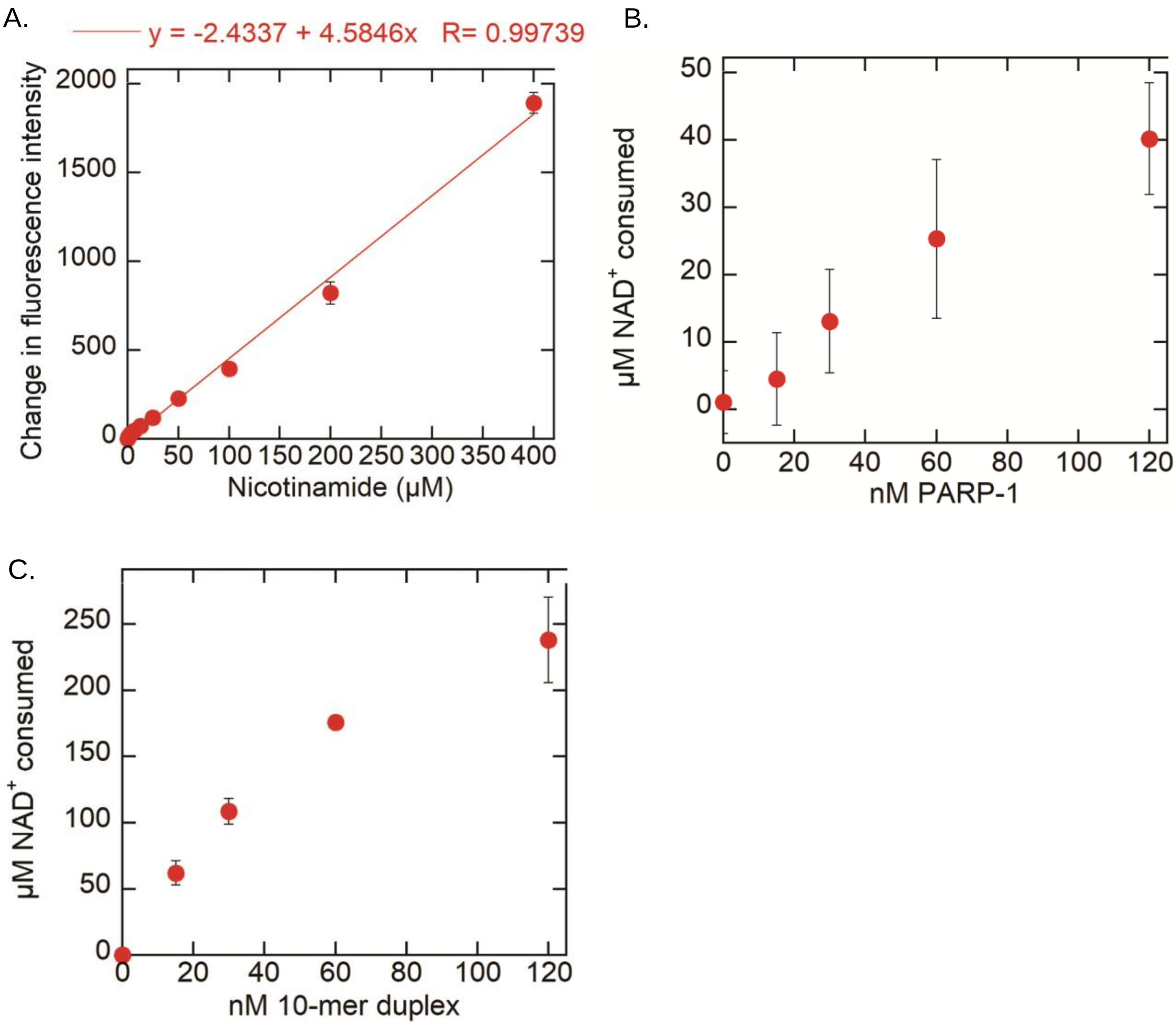

Figure S8. Reproducibility of the yPnc-1 OPT assay. A. Serially-diluted nicotinamide (0-400 µM) was incubated with 20 µg/mL yPnc-1. The net fluorescence was calculated for each sample by subtracting the fluorescence of the control reaction (0 µM nicotinamide) from the experimental reactions. Change in fluorescence intensity was plotted against nicotinamide concentration. The standard curve was fitted to a linear regression. R=0.99739. Error bars indicate standard deviation of triplicate experiments. B. 1 µM 10-mer duplex was incubated with 5 mM NAD^+ and 0-120 nM PARP-1. The auto-modification reactions were quenched with 50 mM benzamide after 15 min followed by addition of 20 µg/mL yPnc-1. NAD^+ consumed in the reaction is plotted for each concentration of PARP-1. Error bars indicate standard deviation of quadruplicate experiments. C. 1 µM PARP-1 was incubated with 5 mM NAD^+ and 0-120 nM 10-mer duplex. The auto-modification reactions were performed similarly to Figure S8B. Error bars indicate standard deviation of triplicate experiments.

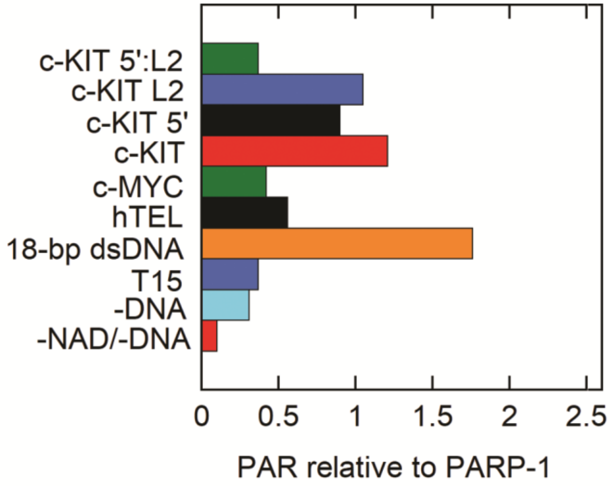

Figure S9. Western Blot quantification of Fig. 5B. Total PAR intensities for each substrate were quantified relative to total PARP-1 using ImageQuant software (GE Healthcare Life Sciences, Pittsburgh, PA).

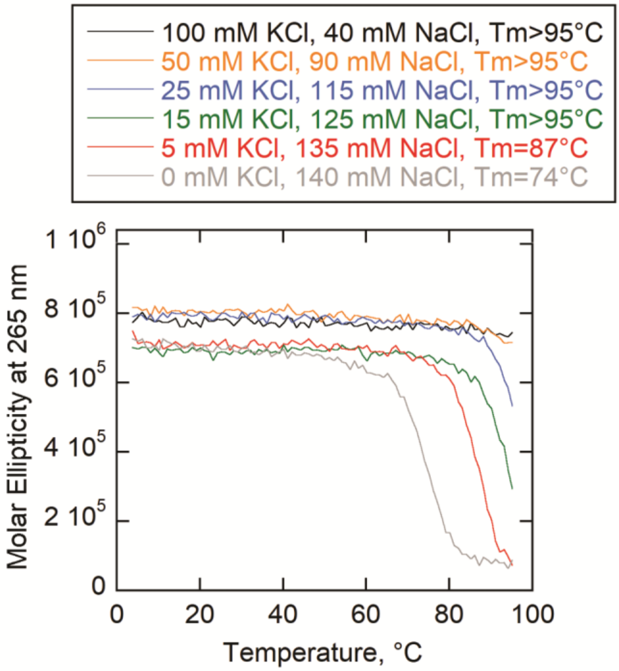

Figure S10. Raw molar ellipticity profile of c-KIT 5':L2 as a function of varying monovalent cation concentrations, Fig. 6B. Molar ellipticity was measured at 265 nm across a temperature range of 4-95ºC.

A.

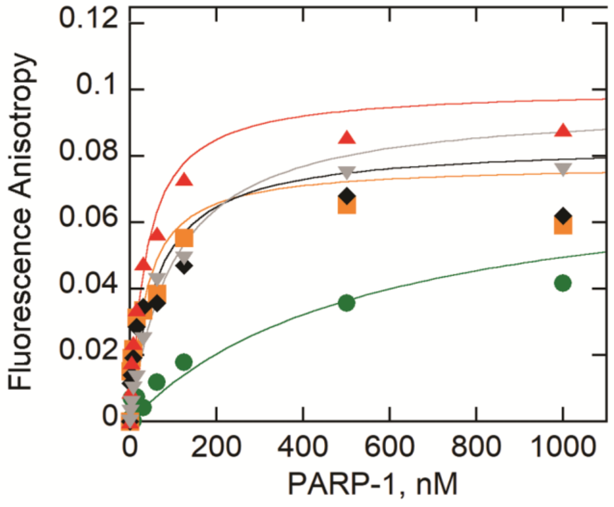

B.

Oligo	K_D (nM)
c-KIT, 140 mM KCl	560
c-KIT 5';L2, 140 mM KCl	40
c-KIT 5':L2, 100 mM KCl, 40 mM NaCl	60
c-KIT 5':L2, 5 mM KCl, 135 mM NaCl	40
c-KIT 5':L2, 0 mM KCl, 140 mM NaCl	100

Figure S11. A. Fluorescence anisotropy of 5 nM c-KIT in 140 mM KCl, c-KIT 5':L2 in 140 mM KCl, c-KIT 5':L2 in 100 mM KCl and 40 mM NaCl, c-KIT 5':L2 in 5 mM KCl and 135 mM NaCl, and c-KIT 5':L2 in 0 mM KCl and 140 mM NaCl incubated with serial dilutions of PARP-1. B. Data were fit to the quadratic equation to obtain K_D values. Experiment performed with 1 replicate.

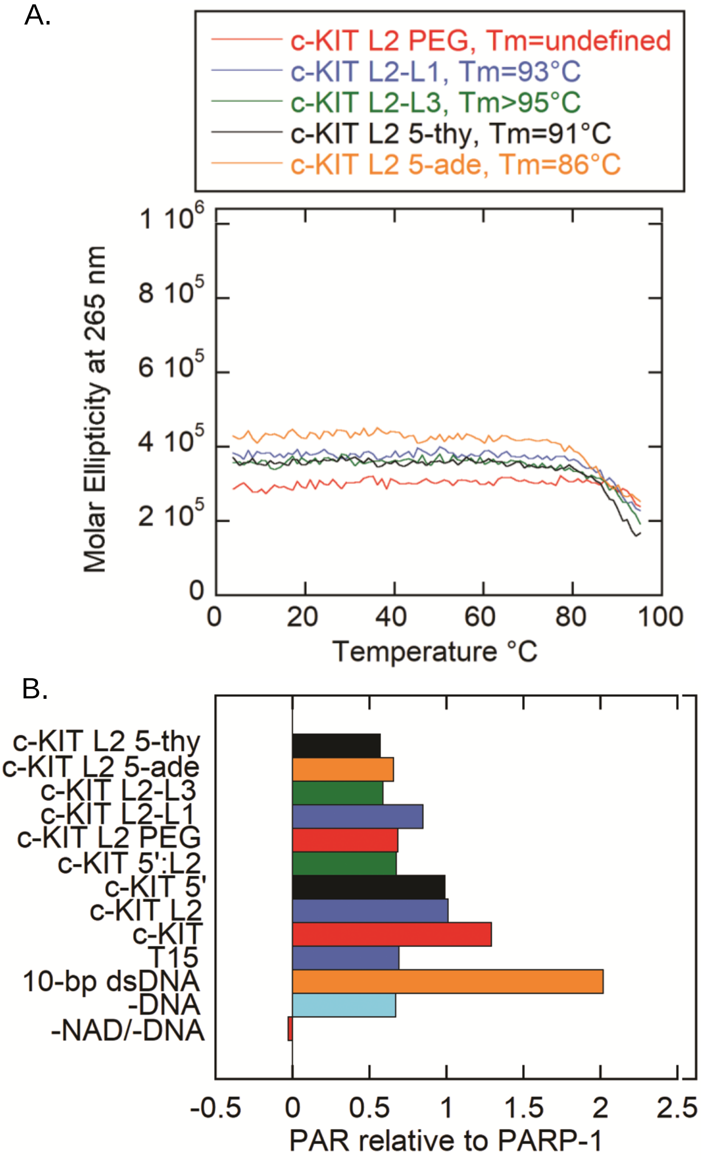

Figure S12. A. Raw molar ellipticity profile of c-KIT G4DNA mutants from Fig. 7B. Molar ellipticity was measured at 265 nm across a temperature range of 4-95ºC. B. Western Blot quantification of Fig. 8B. Total PAR intensities for each substrate were quantified relative to total PARP-1 using ImageQuant software (GE Healthcare Life Sciences, Pittsburgh, PA).

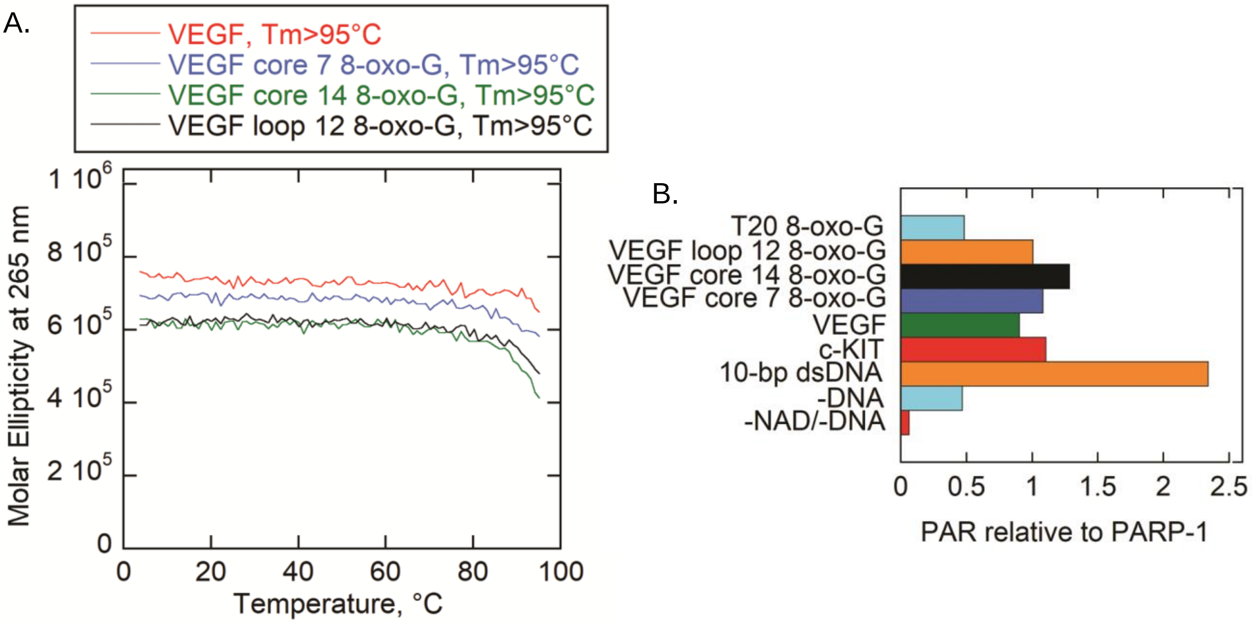

Figure S13. A. Raw molar ellipticity profile of VEGF G4DNA mutants from Fig. 10B. Molar ellipticity was measured at 265 nm across a temperature range of 4-95ºC. B. Western Blot quantification of Fig. 10D. Total PAR intensities for each substrate were quantified relative to total PARP-1 using ImageQuant software (GE Healthcare Life Sciences, Pittsburgh, PA).
